# Amphiregulin normalizes altered circuit connectivity for social dominance of the CRTC3 knockout mouse

**DOI:** 10.1038/s41380-023-02258-x

**Published:** 2023-09-20

**Authors:** Ji-Seon Park, Hwon Heo, Min-Seok Kim, Seung-Eun Lee, Sukyoung Park, Ki-Hyun Kim, Young-Ho Kang, Je Seong Kim, Young Hoon Sung, Woo Hyun Shim, Dong-Hou Kim, Youngsup Song, Seung-Yong Yoon

**Affiliations:** 1 ADEL Institute of Science & Technology (AIST), ADEL, Inc., Seoul, South Korea; 2 Department of Medical Science, Asan Medical Institute of Convergence Science and Technology, Asan Medical Center, University of Ulsan College of Medicine, Seoul, South Korea; 3 Department of Brain Science, Asan Medical Center, University of Ulsan College of Medicine, Seoul, South Korea; 4 Department of Biomedical Sciences, Asan Medical Center, University of Ulsan College of Medicine, Seoul, South Korea; 5 Department of Cell and Genetic Engineering, Asan Medical Center, University of Ulsan College of Medicine, Seoul, South Korea; 6 Department of Radiology, Asan Medical Center, University of Ulsan College of Medicine, Seoul, South Korea; 7 Stem Cell Immunomodulation Research Center (SCIRC), University of Ulsan College of Medicine, Seoul, South Korea

**Keywords:** Neuroscience, Depression

## Abstract

Social hierarchy has a profound impact on social behavior, reward processing, and mental health. Moreover, lower social rank can lead to chronic stress and often more serious problems such as bullying victims of abuse, suicide, or attack to society. However, its underlying mechanisms, particularly their association with glial factors, are largely unknown. In this study, we report that astrocyte-derived amphiregulin plays a critical role in the determination of hierarchical ranks. We found that astrocytes-secreted amphiregulin is directly regulated by cAMP response element-binding (CREB)-regulated transcription coactivator 3 (CRTC3) and CREB. Mice with systemic and astrocyte-specific CRTC3 deficiency exhibited a lower social rank with reduced functional connectivity between the prefrontal cortex, a major social hierarchy center, and the parietal cortex. However, this effect was reversed by astrocyte-specific induction of amphiregulin expression, and the epidermal growth factor domain was critical for this action of amphiregulin. These results provide evidence of the involvement of novel glial factors in the regulation of social dominance and may shed light on the clinical application of amphiregulin in the treatment of various psychiatric disorders.

## Introduction

Cyclic AMP (cAMP) signaling regulates various cellular and physiological processes. In the brain, it is involved in cognition, memory formation, addiction, anxiety, and depression in part through the regulation of phosphorylation-dependent modulation of cAMP-responsive element-binding protein (CREB)-mediated transcriptional activity [1]. Recently, CREB-regulated transcriptional co-activators (CRTCs) were identified as cAMP signaling sensors and mediators of the metabolic state and hormonal signaling; furthermore, it has been shown that the recruitment of CRTCs to the CREB transcription complex was essential for the full activation of cAMP/CREB-mediated transcription [2]. In humans, members of the CRTC family include CRTC1, CRTC2, and CRTC3. CRTC1 is primarily confined in the brain to regulate long-term memory formation, circadian rhythm, and appetite [3–5], while CRTC2 is primarily found in the liver, where it mediates glucagon effects on hepatic glucose production [6, 7]. CRTC3 in adipose tissue attenuates catecholamine-induced lipid mobilization, and cutaneous CRTC3 promotes ultraviolet radiation (UVR)-induced melanogenesis [8, 9].

While many studies have focused on CRTC1 related to neuroplasticity, very limited information is available regarding the biological role of CRTC3 in the brain although CRTC3 is known to be expressed in the brain [10]. CRTC3 is essential for the regulation of CREB-mediated transcription of the corticotropin-releasing factor after a stress stimulus [11]. CRTC3 was recently reported to be related to treatment-resistant depression in genome-wide association study [12] and also as a genetic variant associated with autism spectrum disorders and schizophrenia [9, 13]. A meta-analysis revealed that CRTC3 is significantly associated with chronic postoperative pain implying disorders in the nervous processing of pain [14]. Although CRTC3 was reported to be associated with psychiatric conditions, few studies have studied the intrinsic role of CRTC3 in the brain.

Social dominance trait in individuals is an important factor in establishing social relations, hierarchy, and stability among individuals in society, and even among nations. Although there is growing evidence that various brain areas and networks, including the hippocampus, amygdala, striatum, thalamus, and prefrontal cortex, are associated with social dominance, to date almost no intrinsic neural factors regulating social dominance have been identified [15, 16]. Herein, by addressing the behavioral phenotypes of CRTC3 knockout mice, we found amphiregulin, a transcriptional target of CRTC3, as a novel social hierarchy factor, increasing functional connectivity between the prefrontal and parietal cortex.

## Materials and methods

Detailed materials and methods are described in supplementary information.

### Animals

CRTC3 KO mice, described previously [8], were backcrossed at least 12 times to ensure uniform C57BL6/J genetic background. To compare wild-type (WT) and CRTC3 KO mice, two or three pairs of siblings were separated from their litter and housed together in one cage from birth. Each mouse in the sibling group was randomly selected. Mice with the CRTC3-floxed allele were generated by rederiving the cryopreserved mouse sperm carrying the CRTC3-floxed allele, acquired from the KOMP repository (University of California, Davis, CA, USA). Chimeric mice carrying the CRTC3-floxed allele (CRTC3-fl/+) were crossed with flippase-expressing transgenic mice to remove the neomycin cassette followed by backcrossing with C57BL6/J mice to select mice carrying CRTC3-floxed allele but without flippase. In this experiment, CRTC3-floxed mice, backcrossed to C57BL6/J mice for at least 8 generations, were used. For experiments using C57BL/6 mice, 12-week-old male mice were purchased from Koatech Technology Corporation (Seoul, South Korea). Three pairs of siblings were housed together in one cage from birth. For the resident-intruder test, BALB/c mice were also obtained from Koatech Technology Corporation. Each sibling group of mice was housed in a separate cage and maintained at a constant ambient temperature (22 ± 1 °C) with a 12:12 h light-dark cycle and free access to water and food. For Sprague Dawley rat experiments, 6–7-week-old male rats were purchased from Orientbio (Gyonggi, South Korea). All procedures were approved by the Institutional Animal Care and Use Committee of the Asan Institute for Life Sciences in Seoul, South Korea.

### Osmotic pump implantation

AREG, AREG-EGF, AREG-HB, and mouse EGF proteins were dissolved in phosphate-buffered saline (PBS). Micro-osmotic pumps (flow rate, 0.11 μL/h for 25 d; Alzet model 1004, Charles River, France) were filled with dissolved proteins. The cannula (Alzet brain infusion kit 3) was connected to an Alzet micro-osmotic pump via 4 mm-long vinyl tubing (inner diameter 0.69 mm) filled with dissolved proteins. The brain infusion pumps were placed in a conical tube filled with (PBS) and primed for 48 h at 37 °C before subcutaneous implantation.

Mice were anesthetized with isoflurane (induction: 5%, mask: 2%) and then implanted with a cannula into the right lateral ventricle (anteroposterior: -0.3 mm from bregma, lateral: 1 mm from bregma) using a stereotaxic apparatus. Osmotic pumps were implanted in the back of the mice. At the end of the surgery, mice were administered ketoprofen (8 mg/kg) subcutaneously as post-surgical anti-pain medication. Ketoprofen injections were repeated once the next day. To allow mice recovery, they were not assessed in the tube dominance test for 7 days.

For functional magnetic resonance imaging (fMRI) analysis in rat, micro-osmotic pumps (flow rate of 0.25 μL/h for 28 d, Alzet model 2004) were filled with AREG-EGF and PBS. The stainless-steel cannula was replaced with a 28-gauge polyetheretherketone cannula. Rats (5–6-week-old) were implanted with a cannula into the lateral ventricle (anteroposterior: −1.2 mm from bregma, lateral: −2 mm from bregma, dorsoventricular: 5 mm from the skull using a stereotaxic apparatus. At 21 d after infusion surgery, fMRI analysis was performed, and rats were euthanized the next day.

### Adeno-associated viruses (AAVs) and stereotaxic surgery

Cre-dependent AAV5 and GFAP-AREG-HA-expressed AAV5 viral vectors were cloned and packaged by VectorBuilder (Chicago, IL, USA). Mice at the age of 3–4 months were anesthetized with a zoletil/rompun mixture (4:1) (zoletil 40 mg/kg and rompun 10 mg/kg) and prepared for stereotaxic surgery. For AAV5-GFAP-Cre experiments, a 10 μL Gastight Syringe (Hamilton, 1701RN) was used to inject AAV5s into the brain right ventricle (1 × 10^10^ GC/ mouse, in a total volume of 4 µl, AP: −0.3, ML: −1.0, DV: −3.0 mm). AAV5-GFAP-AREG-HA and control virus solution (1 × 10^11^ GC/mouse in a total volume of 3 µL) were injected into the right ventricle (AP: −0.3, ML: −1.0, DV: −3.0 mm). Viruses were injected at a rate of 0.4 µl/min and the syringe was left in place for an additional 5 min for complete viral vector diffusion. The needle was then slowly removed from the brain for 5 min. For recovery from anesthesia, mice were placed with warm pads to maintain the body temperature. After 1.5–2 weeks, the behavioral tests were performed and all experiments were conducted within 2 months after virus administration. Mice were monitored for general health for 72 h post-surgery.

### Magnetic resonance imaging (MRI) data acquisition

MRI was performed using a 7.0 T Bruker PharmaScan 70/16 MRI system (Bruker BioSpin, Ettlingen, Germany) with ParaVision 6.0.1 software in a configuration involving a 72-mm transmit volume coil and a brain surface receiver coil. For the mouse MRI data acquisition, mice were initially anesthetized with isoflurane (5% for induction and 2% during set-up) in a 20 % O2/80 % air mixture and a bolus of 0.05 mg/kg medetomidine was injected subcutaneously (s.c.). After the mice were secured in a prone position in a Bruker mouse cradle, 0.5% isoflurane was given during the MRI acquisition. For the rat MRI data, rats were anesthetized using 50 mg/kg tiletamine plus zolazepam (Zoletil; Virbac, New South Wales, Australia) and 12.5 mg/kg xylazine (Rompun; Bielkorea, Seoul, South Korea). Respiration was monitored, and the temperature of the rats was maintained at 37.5 ± 0.5 °C using a circulating water bath system (CW-05G heated circulating water bath; MIDSCI, St. Louis, MO, USA). A single-shot gradient-echo echo-planar imaging sequence was used with the following parameters: repetition time (TR) = 1000 ms; echo time (TE) = 20.3 ms; matrix size = 64 × 64; field of view (FOV) = 20×20 mm^2^ (mouse), 25×25 mm^2^ (rat); slice number = 23; slice thickness = 0.6 mm (mouse), 1 mm (rat); slice gap = 0 mm; flip angle = 45°, 600 (mouse), 300 (rat) volumes per scan. Anatomical images were acquired with rapid imaging with a refocused echoes sequence with the following parameters: TR = 4000 ms (mouse), 5250 ms (rat); TE = 33 ms (mouse), 66 ms (rat); matrix size=256×256; FOV = 20×20 mm^2^ (mouse), 25×25 mm^2^ (rat); slice Number=23; slice thickness=0.6 mm (mouse), 1 mm (rat); slice gap=0 mm; and number of excitations (NEX) = 1 (mouse), 2 (rat).

### Resting-state (rs)fMRI data preprocessing

All data were processed using Analysis of Functional NeuroImages (version 19.2.23, National Institutes of Health, Bethesda, MD, USA (29-31) and in-house codes on Matlab (Mathworks, Natick, MA, USA). The preprocessing procedures followed those commonly used in human rsfMRI data but were adapted to optimize the performance of all animal rsfMRI data. The preprocessing pipeline included five steps. First, rsfMRI images were co-registered to animal brain echo-planar imaging template with an affine transformation (3dAllineate). Second, co-registered rsfMRI images were corrected for head motion, and six motion parameters were written to output ASCII format (3dvolreg). Third, motion-corrected rsfMRI images were obtained with the large transients and polynomial trends removed (3dDespike and 3dDetrend). Fourth, nuisance signals were extracted from detrended rsfMRI images using subject-adjusted cerebrospinal fluid (CSF) template mask sets (3dmaskave). Last, detrended rsfMRI images were obtained using bandpass filtering (0.01–0.1 Hz) and spatial smoothing (FWHM = 0.5 mm (mouse), 0.7 mm (rat)) with the nuisance regress out (3dTproject). For the nuisance signal estimate, the six motion parameters and CSF signals were estimated. The region of interest (ROI) of CSF used the CSF mask template from turone mouse brain template and atlas (TMBTA) mouse brain template (33516895) or the SIGMA rat brain template and was modified to include the inside ventricular regions alone. The original TMBTA or SIGMA CSF mask has regions outside the brain area used in this study. Some of the post-registered images were overlaid onto cortical areas outside the region of the original CSF mask. The removal of areas outside the CSF mask was performed using the ITK-SNAP program version 3.6 (16545965). A strict ventricular CSF mask was used in this study. The mean of the time-course signal of the strict CSF mask area was estimated from detrended rsfMRI images (3maskave).

### Resting-state functional connectivity analysis

To measure resting-state functional connectivity (rsFC) in the all animal brains, seed-based rsFC analysis was conducted. To evaluate rodent social dominance behavior-related rsFC, a total of 31 (mouse) 33 (rat) ROIs were assessed (Figs. S13 and S24). Detailed anatomical information of all seed ROIs can be found in Table S1. The averaged time-course signal from each ROI of defined anatomical animal brain regions was based on the TMBTA mouse brain atlas (33516895) for mouse and the SIGMA rat brain atlas for rat and in-house ROI masks whose regions defined were based on the Brain maps 4.0 (29277900). Pearson’s correlation coefficient was used to estimate the rsFC strength across the all animal brains and between ROIs. Subsequently, the correlation values were subjected to a Fisher r-to-z transformation to improve normality. Group-level rsFC maps were generated, which were corrected using family-wise error correction (false discovery rate [FDR] correction; *p* < 0.05). For rat fMRI study, a seed-based spatial FC map was generated with the seed of a 1 mm sphere in the posterior cingulate cortex (24b’ and 24a’). The group-level seed map was calculated using the mean for each of the seeded spatial FC maps (3dTcorr1D, 3dttest + +, and the FDR correction [*p* < 0.05]).

### Statistical analyses

Data were analyzed using Graph Pad Prism 5 software. Data from behavioral tests were analyzed with the Mann-Whitney test, unpaired t-test, and one-way analysis of variance (ANOVA) with post-hoc Bonferroni’s and Turkey’s multiple comparison tests. The Wilcoxon signed-rank test was used to compare pre- and post-drug infusion experiments. Bands from western blotting were analyzed using a t-test or Mann-Whitney test for a two-group comparison where appropriate. When variances were not equal as assessed by the Bartlett test, non-parametric statistics were conducted using the Kruskal–Wallis test followed by Dunn’s multiple comparison test. The D’Agostino & Pearson omnibus normality test was used to test data normality. Probabilities of < 5% (*p* < 0.05) were considered significant. All values are expressed as mean ± standard error of the mean (SEM).

For the rsFC and ChIP assay analyses, an unpaired Student’s t-test was used to compare the differences between the groups. Statistical comparisons were performed with Matlab. In all tests, statistical significance was set at *p* < 0.05. All group-level values are represented as mean ± standard error of the mean (SEM).

## Results

### Low social rank and decreased functional connectivity of prefrontal cortex in CRTC3-knockout mice

**Fig. 1:**
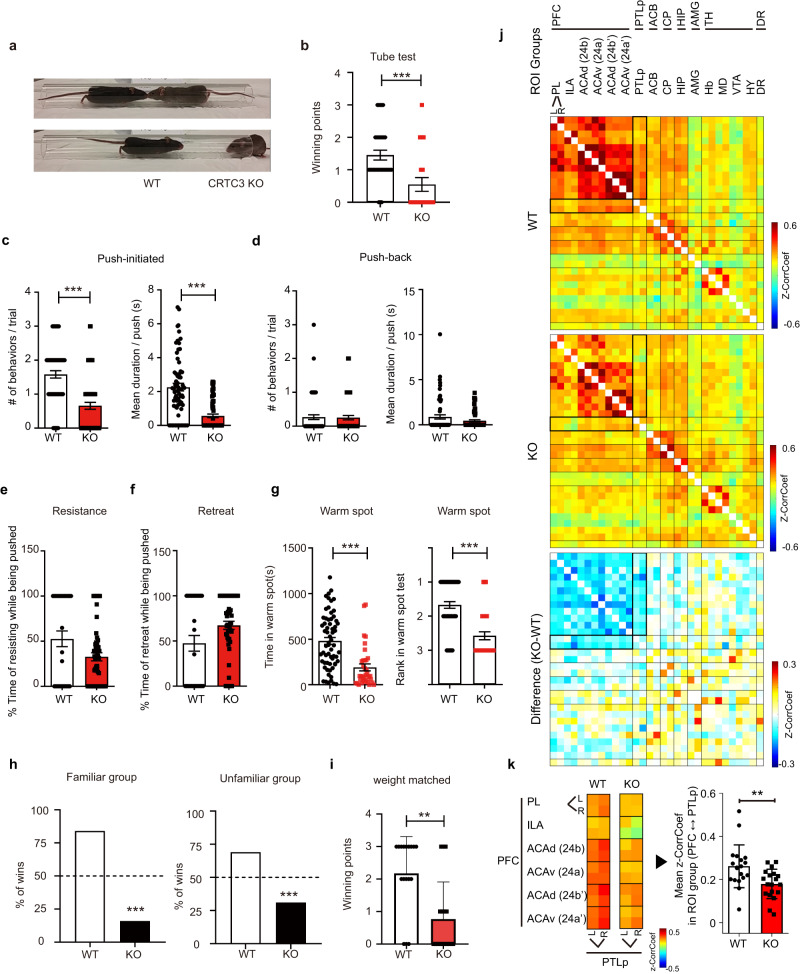
KO mice lacking astrocytic factor CRTC3 show lower social dominance. **a** Tube dominance test. **b** Number of winning points with WT and CRTC3 KO. WT (*n* = 33), KO (*n* = 20). **c**, **d** The number and mean duration of pushes initiated (**c**) and push-backs (**d**), WT (*n* = 67), KO (*n* = 67). Percentage of time that mice resisted while being pushed (**e**) or retreated while being pushed (**f**), WT (*n* = 31), KO (*n* = 58). **g** Time spent in the warm spot (left) and the rank (right) obtained in the warm spot test, WT (*n* = 64), KO (*n* = 36). **h** The percentage of wins in the tube dominance test in familiar groups (left) WT (*n* = 51), KO (*n* = 28) and in unfamiliar groups (right) WT (*n* = 12), KO (*n* = 11). **i** Winning points in the tube dominance test by WT and CRTC3 KO mice of equal weight, WT (*n* = 17), KO (*n* = 17). **j** The resting-state functional connectivity matrices of social dominance-related brain ROIs from WT (left) and CRTC3 KO (middle) mice are represented. The mean difference map is represented (right). **k** Compared with WT, CRTC3 KO mice show significantly decreased mean z-CorrCoef across all focused matrices between the ROI groups of the prefrontal cortex (PFC) and posterior parietal cortex (PTLp). L: left and, R: right hemispheres. WT (*n* = 19), KO (*n* = 19), Mann-Whitney test, Chi-square, Unpaired t-test. **p < 0.01, ***p < 0.0001. Error bars, SEM.

To explore the CRTC3 function in the brain, we conducted neurobehavioral tests and found that CRTC3-knockout (CRTC3 KO) mice exhibited significantly fewer wins than control WT mice in the dominance tube test (Fig. 1a, b, S video 1). CRTC3 KO mice initiated significantly fewer pushes and showed a shorter duration per push than control WT mice (Fig. 1c). While there was no difference in the number and mean duration of push-backs between CRTC3 KO and WT mice (Fig. 1d), when CRTC3 KO mice were pushed, although statistics did not reach significance, they tended to be less resistant (Fig. 1e) and retreated more often than WT mice (Fig. 1f). Furthermore, supporting the lower social hierarchical ranking of the CRTC3 KO mice in the dominance tube test, CRTC3 KO mice spent less time in the warm spots than control WT mice and ranked a lower hierarchy in the warm spot competition test (Fig. 1g). Moreover, CRTC3 KO mice won significantly less often against both familiar and unfamiliar aged-matched WT mice (Fig. 1h) as well as against weight-matched WT mice (Fig. 1i), indicating that the lower hierarchical ranking of the CRTC3 KO mice were due to a loss of the *CRTC3* gene. Regarding gender-specific issues, CRTC3 KO female mice also showed lower hierarchical ranking than WT mice, similar to male mice (Fig. S1). CRTC3 KO mice displayed fewer urine spot areas than WT mice (Fig S2), ensuring the lower social rank of CRTC3 KO mice. Arguing against nonspecific effects of CRTC3 on neurobehavioral changes, the brain development assessed using Nissl-stained brain sections of CRTC3 KO appeared to be comparable to that of WT mice (Fig. S3), and no significant differences were observed between CRTC3 KO and WT mice in memory formation (Fig. S4), mood (Fig. S5), strength (Fig. S6), and sensory ability (Fig. S7) tests. These results suggest that CRTC3 plays a specific role in determining the social hierarchical ranking in mice.

To investigate whether lower social dominance ranks in CRTC3 KO mice were associated with alterations in functional brain networks, we performed fMRI to examine whether brain regions and functional connectivity were affected by CRTC3 deficiency. Analysis of the correlation coefficient (CorrCoef) of resting-state blood-oxygen-level-dependent (BOLD) signals of 33 ROIs related to social dominance behaviors revealed that CRTC3 KO mice exhibit decreased rsFC in the prefrontal cortex (PFC) and posterior parietal association areas (PTLp) regions (Fig. 1j). The mean PFC-PTLp CorrCoef was decreased by 80% in the CRTC3 KO group compared with that of WT (*p* = 0.0099, Fig. 1k).

### CRTC3 is mainly expressed in astrocytes

**Fig. 2:**
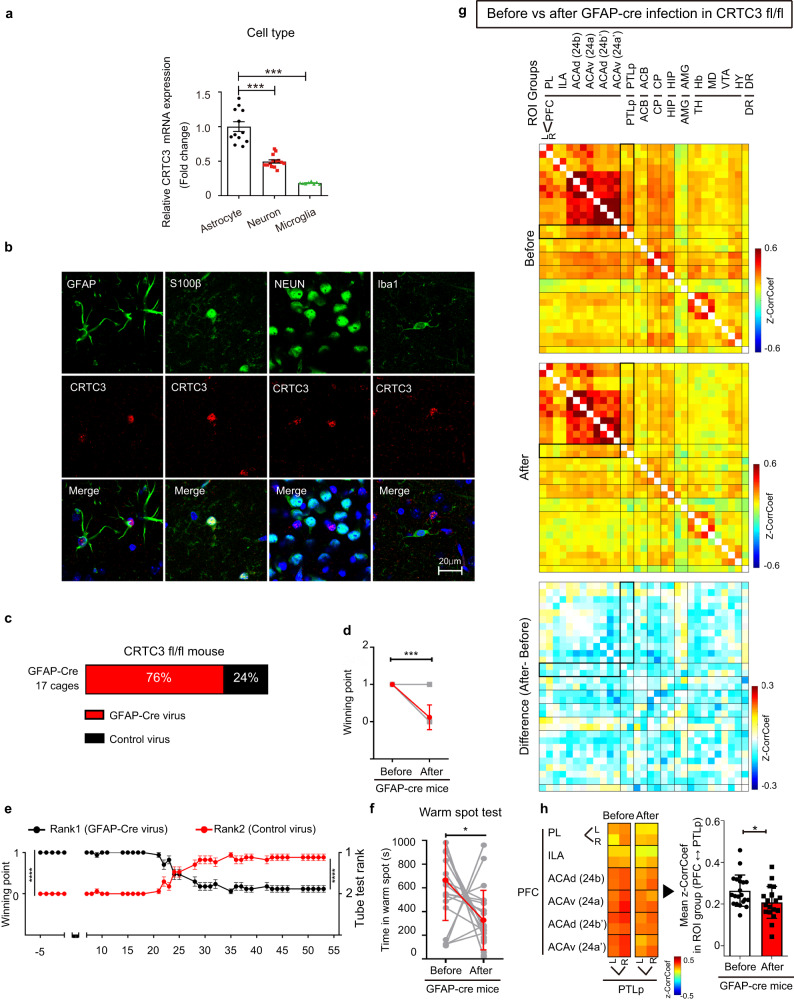
CRTC3 is mainly expressed in astrocytes. **a** Relative *CRTC3* mRNA expression in primary cell cultures. One-way ANOVA, astrocytes (*n* = 12), neurons (*n* = 14), microglia (*n* = 8). **b** Representative immunohistochemistry co-stained with antibody against CRTC3 (red) and various cell markers (green). **c** Percentage of cages with mice showing the change in the rank after administration of GFAP-Cre virus in CRTC3 fl/fl mice, 17 cages. **d** Changes in the winning points by each mouse (gray), and the average winning points (red) before and after GFAP-Cre virus infection in CRTC3 fl/fl mice, before (*n* = 17), after (*n* = 17). **e** Summary of changes in winning points after infusion, rank1 (*n* = 17), rank2 (*n* = 17). **f** Time spent in the warm spot (right) by each mouse (gray), and the average points (red) before and after GFAP-Cre infection virus in CRTC3 fl/fl mice, before (*n* = 20), after (*n* = 20). **g** The resting-state functional connectivity matrices of social dominance-related brain ROIs from control (left) and GFAP-Cre (middle) post-injection states are represented. The mean difference map is represented (right). **h** Compared with the pre-injection state, GFAP-Cre AAV5-infected CRTC3 fl/fl mice show significantly decreased mean z-CorrCoef across all focused matrices between the ROI groups of the prefrontal cortex (PFC) and posterior parietal cortex (PTLp), before (*n* = 20), after (*n* = 21). ANOVA test (Tukey’s multiple test), Wilcoxon signed rank test, Kruskal-Wallis test, Unpaired t-test, *p < 0.05, ***p < 0.001, **p < 0.0001.

While it is well-known to be abundant in adipose tissues, CRTC3 expression in the brain remains unknown. Immunofluorescence staining of the mouse brain sections for CRTC3 indicated that this protein, rather than being restricted to specific brain regions, appeared to be broadly expressed in the central nervous system including the cortex, hippocampus, amygdala, basal nuclei, and brain stem (Fig. S8). CRTC3 KO mice showed no immunoreactivity, confirming the specificity of the antibody (Fig. S9). To identify the types of cells that mainly expressed CRTC3 in the brain, we cultured primary mouse neurons, astrocytes, and microglia cells and compared the relative amount of *CRTC3* mRNA among these cells. qRT-PCR analysis of *CRTC3* mRNA showed that astrocytes had the highest expression level of CRTC3 in comparison to neurons or microglia (Fig. 2a). Similar to observations in adipocytes, CRTC3 in astrocytes also existed in a phosphorylated form in the basal state and was dephosphorylated in response to cAMP stimulation (Fig. S10). Consistent with the abundant expression of CRTC3 in cultured primary astrocytes, CRTC3 immunoreactive cells in mouse brain sections were primarily co-localized with astrocytes (as shown using Glial fibrillary acidic protein (GFAP) and S100β markers for immunoreactive cells) but not with neurons (NeuN to indicate immunoreactive cells) and microglia (Iba1 to label immunoreactive cells) (Fig. 2b).

### Low social rank and decreased functional connectivity of PFC in astrocyte-specific CRTC3 KO mice

To test whether CRTC3-deficiency in astrocytes is responsible for the behavioral phenotype observed in CRTC3 KO mice, we knocked out CRTC3 specifically in astrocytes by administering AAV5 expressing Cre recombinase under the regulation of GFAP promoter in the ventricles of CRTC3-fl/fl mice (Figs. S11 and S12). Similar to systemic CRTC3 KO mice, astrocyte-specific CRTC3-knockout (CRTC3fl/fl-GFAP-Cre) mice exhibited significantly fewer wins than control WT mice in the dominance tube test (Fig. 2c–e, S video 2). Furthermore, supporting the lower social hierarchical ranking of the GFAP-Cre mice in the dominance tube test, GFAP-Cre mice spent less time in the warm spots than control WT mice and ranked a lower hierarchy in the warm spot competition test (Fig. 2f). These results confirm that astrocytic CRTC3 plays a specific role in determining the social hierarchical ranking in mice. To investigate whether brain networks are regulated by CRTC3 in astrocytes, fMRI was performed in astrocyte-specific CRTC3 KO (CRTC3fl/fl-GFAP-Cre) mice (Fig. S13). The CorrCoef of resting-state BOLD signal analysis confirms that GFAP-Cre virus injection-mediated astrocyte CRTC3 downregulation decreased rsFC in the PFC and PTLp regions (Fig. 2g), whereas it was not changed after control virus injection (Fig S14). The mean PFC-PTLp CorrCoef was decreased by 70% in the GFAP-Cre group compared with the WT group (*p* < 0.05, Fig. 2h). The PFC-PTLp CorrCoef was also analyzed to be decreased in GFAP-Cre transduction compared to control transduction (Fig. S15).

### AREG is a transcriptional target gene of CRTC3

**Fig. 3:**
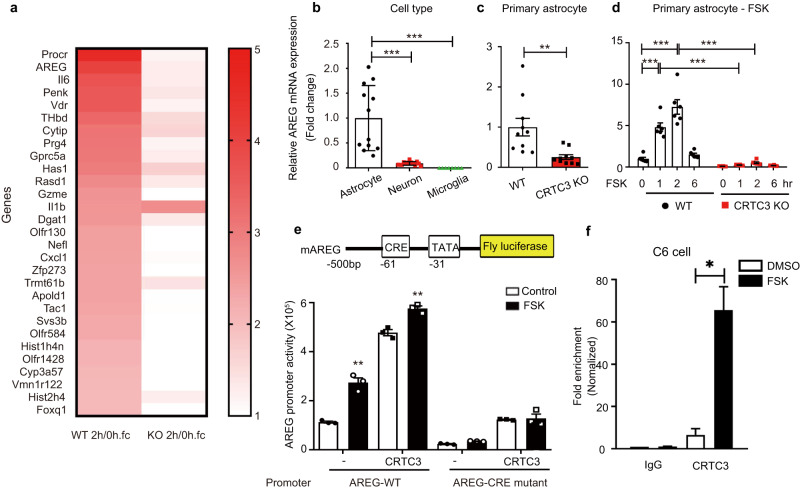
Amphiregulin is a CREB target gene regulated directly by CRTC3. **a** Heat map showing fold-change values in gene expression after 2 h of treatment with forskolin (FSK) in astrocytes. **b** Relative *AREG* mRNA expression in primary cell cultures. astrocytes (*n* = 12), neurons (*n* = 14), microglia (*n* = 8). **c** Relative amphiregulin (AREG) mRNA expression in astrocytes from WT and CRTC3 KO mice, WT (*n* = 10), KO (*n* = 10). **d** Relative AREG mRNA expression in astrocytes after FSK treatment, WT (*n* = 6), KO (*n* = 6). **e** A schematic diagram (top). A luciferase assay of AREG promoter activity (bottom), All samples (*n* = 6). **f** Chromatin immunoprecipitation (ChiP) assay showing occupancy of CRTC3 over the AREG promoter in astrocytes exposed to FSK as indicated, One-way ANOVA, Unpaired t-test, Two-way ANOVA (Tukey’s multiple test), *p < 0.05, **p < 0.01, ***p < 0.001 Error bars, SEM.

After confirming the effects of astrocyte CRTC3 on social dominance and functional brain connectivity, we hypothesized that the loss of astrocyte CRTC3 affects neurons and causes behavioral changes in CRTC3 KO mice. To identify astrocyte-derived factors that were regulated by CRTC3 and responsible for altering social dominance behaviors and functional neural networks, primary astrocyte cultures of WT and CRTC3 KO mice were treated with vehicle or FSK, and the genome-wide transcriptome profile was analyzed. We speculated that genes with high cAMP responsiveness in WT cells but lower or missing responsiveness in CRTC3 KO cells were transcriptional targets of CRTC3. Heatmaps, analyzed by this standard, revealed genes involved in multiple signaling pathways, including the Gprotein-coupled receptor signaling pathway, inflammation, homeostasis, and glial proliferation, as potential targets for CRTC3 in astrocytes (Fig. 3a). *AREG* was one of the highest-ranked genes and was an autocrine or paracrine factor that is secreted extracellularly and may affect neurons and their networks. AREG is a member of the EGF family that is synthesized as a type I transmembrane glycoprotein [17, 18]. Upon release through proteolytic cleavage by tumor-necrosis factor-alpha converting enzyme or a disintegrin and metalloproteinase 17 (ADAM17), AREG modulates cell proliferation, mammary ductal morphogenesis, inflammation, and neural cell division [19–21]. Similar to the CRTC3 expression pattern, AREG also appears to be highly enriched in astrocytes in comparison with neurons and microglia (Fig. 3b, S16–17) and consistent with the transcriptome analysis, the mRNA level of *AREG* was lower in astrocytes of CRTC3 KO than in WT mice (Fig. 3c). We then evaluated whether the *AREG* gene was a direct target of cAMP and CRTC3 in astrocytes. In parallel with the burst-attenuation kinetics of CRTC3/CREB target gene expression, *AREG* mRNA levels in WT primary astrocytes peaked within 1–2 h after cAMP stimulation and gradually decreased, whereas that kinetics of *AREG* mRNA expression was blunted in CRTC3 KO astrocytes (Fig. 3d). In line with the presence of a conserved cAMP response element (CRE) site at -61 and a TATA box at -31 of the *AREG* gene, FSK exposure resulted in upregulated AREG promoter activity, which was further enhanced by CRTC3 overexpression, whereas CRE mutant AREG promoter activity was not stimulated by FSK and CRTC3 overexpression (Fig. 3e). Moreover, recruitment of CRTC3 to the AREG promoter around the consensus CRE site was further increased in astrocytes exposed to cAMP as observed using ChIP analysis (Fig. 3f, Fig. S18). These results demonstrate that AREG is primarily produced in astrocytes, and its expression is directly regulated by CRTC3.

### AREG is a determining factor for astrocyte CRTC3-regulated social ranking

**Fig. 4:**
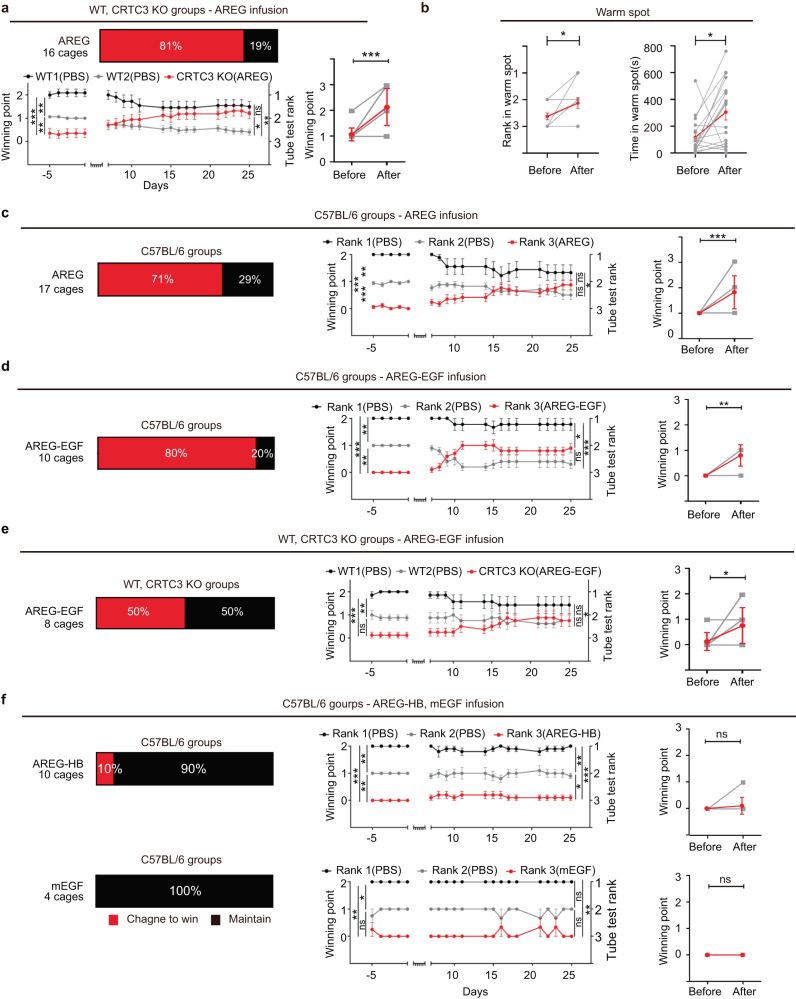
Infusion of AREG and the EGF domain improves social rank in WT and KO mice. **a** Percentage of cages with mice showing the change in rank after infusion of AREG in CRTC3 KO mice (Up), 16 cages. Summary of changes in winning points after infusion (down, left), WT1 (*n* = 11), WT2 (*n* = 17), KO (*n* = 17), Changes in the winning points by each mouse (gray), and the average winning points (red) before and after AREG infusion in CRTC3 KO mice. (down, right), before (*n* = 16), after (*n* = 16). **b** Rank (left) and time spent in the warm spot (right) by each mouse (gray), and the average points (red) before and after AREG infusion in CRTC3 KO mice, before (*n* = 16), after (*n* = 16). **c**–**f** Summary change of the tube dominance test in AREG infused-WT mice groups, Rank1 (*n* = 9). Rank2 (*n* = 17), Rank3 (*n* = 1)7, before (*n* = 23), after (*n* = 23) (**c**), AREG-EGF infused-WT mice groups, Rank1 (*n* = 9), Rank2 (*n* = 10), Rank3 (*n* = 10), before (*n* = 10), after (*n* = 10) (**d**), AREG-EGF infused CRTC KO mice group, WT1 (*n* = 7), WT2 (*n* = 13), KO (*n* = 13), before (*n* = 8), after (*n* = 8) (**e**) and AREG-HB or mEGF-infused WT mice groups, AREG-HB; AREG-HB Rank1 (*n* = 10), Rank2 (*n* = 10), Rank3 (*n* = 10), before (*n* = 10), after (*n* = 10), mEGF; Rank1 (*n* = 4), Rank2 (*n* = 4), Rank3 (*n* = 4), before (*n* = 4), after (*n* = 4) (**f**). Kruskal-Wallis test, Wilcoxon matched-pairs signed rank test, ns, *p < 0.05, **p < 0.01, ***p < 0.001. Error bars, SEM.

To determine whether the low social hierarchical ranking of CRTC3 KO mice was due to downregulation of AREG, we inserted an osmotic pump into the right lateral ventricle of the mouse brain, and vehicle and amphiregulin were introduced into control WT and CRTC3 KO mice, respectively. Consistent with our previous observations in the dominance tube test, the initial hierarchical ranking of CRTC3 KO mice was lower than that of the control WT mice and this was significantly increased by amphiregulin infusion (Fig. 4a). CRTC3 KO mice infused with amphiregulin exhibited an increased number of push-initiation, push duration, and positive modulation of resistance behavior (Fig. S19a); furthermore, overall, in 81% of the tested cages, the lower dominant trait due to CRTC3 deficiency was reversed by AREG treatment (Fig. 4a, S video 3). In line with this, the time spent in the warm spot and the ranking of CRTC3 KO mice in the warm spot test were also increased after AREG infusion (Fig. 4b). Regarding the specificity of the AREG effect, there was no change in the hierarchical ranking observed in the cages with vehicle-treated CRTC3 KO mice (Fig. S20). Having observed the amphiregulin effect on CRTC3 KO mice, we hypothesized that AREG could modulate social dominance behavior in WT mice. Therefore, we performed dominance tube tests in WT mice to determine hierarchical ranking among them; subsequently, the dominance tube tests were resumed after administering AREG to the lowest-ranked WT mice and vehicle to the remaining upper-ranked WT mice. Similar to the observations in CRTC3 KO mice, infusion of AREG led to a change in the behavior of the lowest-ranked WT mice toward winning behavior, such that 71% of the cages showed disruption in the previous stable hierarchy (Fig. 4c). Over time, the lowest-ranked mice, which were infused with AREG, tended to beat the previously middle-ranking mice, winning more points, and thus altering the distinct ranks previously observed in their social hierarchy (Fig. 4c). Moreover, AREG elevated social ranking by increasing the initiation of dominance displays (push-initiation), push duration, and the tendency to hold ground against the opponent’s aggression (resistance) (Fig. S19b).

### Amphiregulin raises social rank via its EGF domain

Mature AREG comprises the N-terminal of the hydrophilic HB domain and the C-terminal of the EGF domain [17]. These are conserved among various species (Fig. S21a). To determine the specific domain of AREG that mediated the social hierarchy, we synthesized AREG-EGF and AREG-HB domains (Fig. S21b) and evaluated their abilities to increase social ranking. Infusion of AREG-EGF to the lowest-ranked mice from cages of WT mice induced changes to tube dominance test results in 80% of cages (Fig. 4d). Similarly, AREG-EGF infusion into CRTC3 KO mice also disrupted the previously established hierarchy of the cages (Fig. 4e). However, infusion of AREG-HB induced changes in mice in 10% of the cages, while the infusion of mouse EGF (mEGF) did not induce any behavioral change (Fig. 4f). Furthermore, the number of winning points was increased in AREG-EGF-infused mice (Fig. 4e), but not in AREG-HB-infused mice or mEGF-infused mice (Fig. 4f). These data suggest that the EGF domain of AREG is essential for controlling the social dominance ranking.

### Astrocytic expression of AREG-EGF raises the social ranking and increases the functional connectivity of PFC with PTLp

**Fig. 5:**
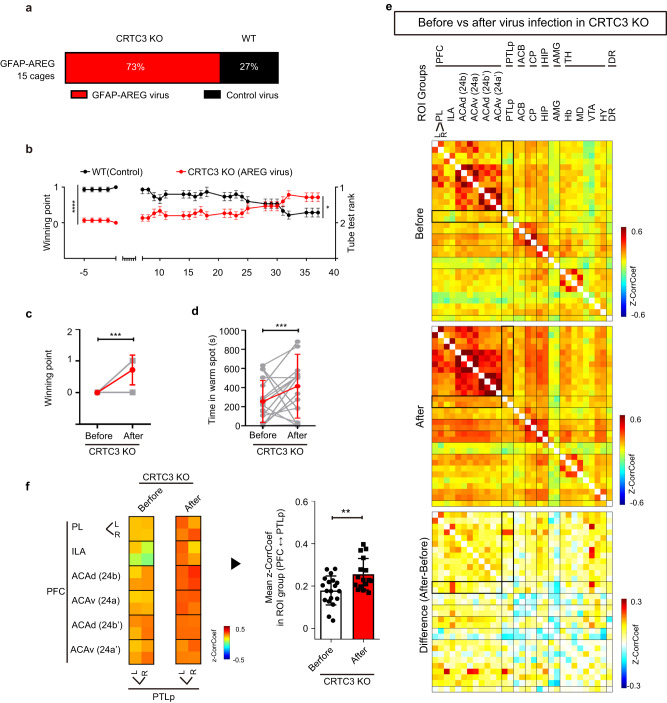
Astrocytic expression of amphiregulin raises social rank and increases functional connectivity. **a** Percentage of cages with mice showing the change in rank after administration of control or GFAP- AREG AAV5 virus in WT and CRTC3 KO mice, 15 cages. **b** Summary of changes in winning points after infusion, WT (*n* = 15), KO (*n* = 15). **c** Changes in the winning points by each mouse (gray), and the average winning points (red) before and after GFAP- AREG virus infection in CRTC3 KO mice, before (*n* = 15), after (*n* = 15). **d** Time spent in the warm spot (right) by each mouse (gray), and the average points (red) before and after GFAP-AREG virus infection in CRTC3 KO mice, before (*n* = 17), after (*n* = 17). **e** The resting-state functional connectivity matrices of social dominance-related brain ROIs from baseline (left) and GFAP-AREG injection states (middle) in CRTC3 KO mice are represented. The mean difference map is represented (right). **f** Compared with baseline, GFAP-AREG virus-infected CRTC3 KO mice show also significantly increased mean z-CorrCoef between the ROI groups of the prefrontal cortex (PFC) and posterior parietal cortex (PTLp). Before (*n* = 19), after (*n* = 16), Kruskal-Wallis test, Wilcoxon signed rank test, Unpaired t-test, *p < 0.05, **p < 0.01, ***p < 0.001, ****p < 0.0001, Error bars, SEM.

Having confirmed the effect of AREG administration on the changes in the hierarchical ranking of mice, we studied whether astrocyte-specific expression of AREG was sufficient for these functions. The hierarchical ranking of CRTC3 KO mice, which initially had a lower hierarchical ranking than WT control mice, was significantly increased by the administration of AAV5 encoding AREG under the control of a GFAP promoter in the ventricles of CRTC3 KO mice (Fig. 5a–c, S22). The time spent in the warm spot and ranking of CRTC3 KO mice in the warm spot test were also increased in CRTC3 KO mice with AREG-induced expression in astrocytes (Fig. 5d). To further investigate whether brain networks are also normalized by AREG-induction in astrocytes, fMRI was performed in CRTC3 KO mice transduced with AAV5 encoding GFAP-AREG and WT mice infected with control AAV5 virus. Administration of GFAP-AREG virus in CRTC3 KO mice increased rsFC and mean CorrCoef in the PFC and PTLp regions compared to the CRTC3 KO baseline, before virus administration (Fig. 5e, f). Contrary to the decrease in PFC and PTLp regions in CRTC3 KO mice (Fig. 2j and k), GFAP-AREG virus administration increased rsFC and the mean CorrCoef to similar levels in WT mice (Fig. S23). Finally, AREG-EGF also increased rsFC and mean CorrCoef in the PFC and PC in rat (Fig. S24 and S25). These results further confirm that astrocytic CRTC3 plays a specific role in determining the social hierarchical ranking in mice as well as in rat via AREG.

## Discussion

Characterization of systemic CRTC3 null mice has made a significant contribution to revealing the physiological role of CRTC3 in energy metabolism, skin melanogenesis, and hematopoiesis [8, 22, 23]. The role of CRTC3 in metabolism was further validated with a tissue-specific CRTC3 knockout mouse model [24]. Applying a similar approach, the present study uncovered an unexpected role for CRTC3 in determining the social hierarchical ranking. We found that CRTC3 is predominantly expressed in astrocytes of various brain areas and plays a fundamental role in determining social ranking by regulating the expression of AREG, a direct transcriptional target gene of CRTC identified in this study. Furthermore, mice with astrocyte-specific downregulation of CRTC3 (Fig. 2) phenocopied the behavior traits observed in systemic CRTC3 knockout mice (Fig. 1) demonstrating that astrocyte CRTC3 was responsible for the altered social behavior traits.

Overlapping expression of CRTC3 and other CRTC family members in multiple tissues has been reported. However, phenotypic analysis of individual KO mice suggested that the function of CRTC1, 2, and 3 in the liver, fat, and skin tissues is independent or that one gene plays a dominant and specific role in a specific tissue [7, 8, 23, 25]. In addition to CRTC3, other CRTC family members, particularly CRTC1, are highly expressed in the brain. CRTC1 KO mice are hyperphagic due to the downregulation of CART expression, whereas there is no change in energy intake of CRTC3 KO mice [3, 8]. Similar but not identical to CRTC3 KO mice, CRTC1 KO mice also exhibited depressive-like behavior but also with severe aggression [26, 27], which was not seen in CRT3 KO mice. These results suggest that the distribution of CRTC1 and 3 expression overlaps but could also be distinctive in the brain and that even at overlapped expression sites, they may have independent functions by regulating the expression of their specific target genes. It would be interesting to generate astrocyte- or neuron-specific CRTC1 null mice and investigate whether the depressive behaviors observed in CRTC1 KO mice are due to AREG downregulation by CRTC1 deficiency in astrocytes or by a completely different mechanism.

Regarding whether AREG is an executor of CREB-mediated regulation of dominant/submissive traits, AREG transcripts are directly regulated by CRTC3 in astrocytes, its mRNA level is downregulated in astrocytes of CRTC3 KO, and the lower hierarchical ranking of CRTC3 knockout mice was reversed by intracerebroventricular administration of AREG peptide and AAV5s encoding AREG. The increase in the social hierarchy by AREG was mediated by its EGF domain (Fig. 4). Since AREG is a member of the EGF family including EGF, TGFα, HB EGF, betacellulin, and epiregulin, we checked whether the increase of social hierarchy by AREG is a common phenomenon induced by EGF family. However, mEGF could not increase the social ranking (Fig. 4f), suggesting that the increase in the social hierarchy is a specific phenomenon induced by AREG, not commonly induced by other EGF family members. AREG has a difference in the amino acids of the receptor-binding domain, which lowers AREG affinity to EGFR compared to the high affinity of other EGF family members [28]. This lower affinity of AREG to EGFR results in tonic activation of downstream signals, distinct from oscillating signals induced by other EGF family members [28]. This molecular difference may explain AREG’s specific effect on social hierarchy (Figs. 4 and 5). Further studies using in vivo mouse genetic models such as astrocyte-specific AREG knockout or overexpressing mice and investigation of downstream signaling will clarify molecular details in the regulation of social behavior.

Although several pathways controlling social hierarchy have been revealed, including the regulation of α-Amino-3-hydroxy-5-methyl-4-isoxazolepropionic acid (AMPA)-type glutamate receptors in medial PFC [15, 29], our findings demonstrated a novel molecular basis of social dominance in mice originating in astrocytes. Astrocytes are the most abundant cells in the brain and are known to play an important role in regulating synaptic activity, affecting various mouse behaviors. For example, astrocytes in the hippocampus modulate memory performance by long-term potentiation or long-term depression [30, 31]. Astrocytes in the arcuate nucleus of the hypothalamus inhibit food uptake by inactivating agouti-related peptide neurons [32]. As such, astrocytes have been reported to be involved in various behaviors, however, little is known about whether astrocytes can affect dominant behavior. Our findings reveal a novel role for astrocytes in establishing a social hierarchy, in which CRTC3 plays a key role by directly regulating AREG production.

PFC is a key area of social hierarchy [33]. Dorsomedial PFC activation initiates and maintains more effortful behaviors in social competition, suggesting a neurobiological foundation for hierarchy-associated traits provided by PFC-based cognitive processes [33]. The structural connection between the rat medial PFC and posterior parietal cortex regions [34] and the importance of frequency-specific coupling of Cg1/2 and posterior parietal cortex regions in the processing of sensory information [35] are well-known. The increased rsFC between the PFC and PTLp regions induced by AREG administration suggests that synaptic reinforcement caused by AREG-induced synchronized BOLD signal patterns in both brain regions (Fig. 5). A human fMRI study showed that the PFC and intraparietal sulcus were important for perceiving social hierarchy [36, 37]. Our results strengthen the notion that the connection of the PFC to the PTLp is the circuit of social hierarchy that can be regulated by AREG.

Behaviors that underlie social hierarchy, and *CRTC3* and *AREG* genes are highly conserved in mammals as also observed in rat (Fig. S24, S25); therefore, we may expect the corresponding molecular basis to be conserved in humans as well. The strive for dominance is prevalent in animals displaying social behaviors. The consequence of social dominance affects not only the possession of sustenance and territories and the selection of partners but also stress levels and mental health; therefore, improving social status using amphiregulin treatment may have a potential therapeutic value. Given that AREG treatment modulates the frontoparietal pathway and noting that CRTC3 and amphiregulin are associated with mental disorders affecting how the brain perceives the world, such as schizophrenia [13, 38, 39], autism [40], as well as mood depressive disorder [12], our findings suggest a new approach to treating such psychiatric disorders either as disease-modifying (restoring perturbed brain function) or symptom-alleviating (alleviating anti-social or dissocial symptoms) treatments.

### Supplementary information

Supplementary material

Supplementary Video S1

Supplementary Video S2

Supplementary Video S3

## Data Availability

The transcript data of primary astrocytes from WT and CRTC3 KO is available in a public database with its accession number GSE222741. All other data are available in the main text and supplementary materials. The datasets and materials generated during and/or analyzed during the current study are available from the corresponding authors (Youngsup Song and Seung-Yong Yoon) on reasonable request.
