# Supplementary material for: Amphiregulin normalizes altered circuit connectivity for social dominance of the CRTC3 knockout mouse
Source: Mol Psychiatry. 2023 Sep 20;28(11):4655–65. doi: 10.1038/s41380-023-02258-x (PMC10914624; doi:10.1038/s41380-023-02258-x)
Supplement: Supplementary file 1 — Supplementary material [file 41380_2023_2258_MOESM1_ESM.docx]

**Suplemmentary Information**

Supplementary Methods.

Supplementary Figures. S1–S25

Supplementary Tables. S1-S2

Supplementary Videos. S1–S3

**Supplementary Methods**

**Reagents**

Recombinant mouse amphiregulin (AREG) and epidermal growth factor (EGF) were purchased from R&D Systems (Minneapolis, MN, USA). AREG-EGF and AREG-heparin-binding (HB) peptides were obtained from PepTron Inc. (Daejeon, South Korea). Forskolin (FSK, 25μM; Sigma-Aldrich, St Louis, MO, USA) was prepared using dimethyl sulfoxide (DMSO).

**Cell culture and isolation of primary cells**

For cultures of primary cortical neurons, the cerebral cortex was isolated from the brains of mouse embryo on embryonic day 16. Cortices were dissected to remove the meninges in cold calcium- and magnesium-free Hanks’ balanced salt solution. After dissection, minced dissected tissue was incubated with a 0.05% trypsin-EDTA for 15 min at 37 °C, and the tube was inverted every 5 min. Trypsin was inactivated with Dulbecco's Modified Eagle Medium containing 20% fetal bovine serum, and the sample was centrifuged at 300g for 15 min. The pellet was gently triturated using a Pasteur pipette. Dissociated cells were mixed with neurobasal medium supplemented with Gibco B-27 components (Life Technologies/ Thermo Fisher Scientific, Grand Island, NY, USA) and added to plates at the required 2.4 x 10^6^ cell density. The plates were coated with poly-d-lysine (Sigma-Aldrich) and laminin (Life Technologies/Thermo Fisher Scientific). Neurons were maintained at 37 °C in a humidified 5% CO_2_ environment. All animal protocols used in this study were approved by the Asan Institute for Life Sciences Animal Care and Use Committee.

For glial cell cultures, the cerebral cortex was isolated from mouse pups on postnatal days 1–3. Glial cells were isolated using similar methods to those used for primary neurons, dissected tissue was incubated with 0.05% trypsin-EDTA for 15 min at 37°C. Cells were centrifuged for 5 min at 300g and resuspended in complete media containing Granulocyte-macrophage colony-stimulating factor (5 ng/mL). Resuspended cells were plated in a T75 flask and incubated at 37°C for 10 days. For isolation of primary microglia, mixed glial cells were collected by shaking the flask at 225 rpm for 2 h. Floating primary microglial cells were collected and plated in a poly-d-lysine-coated plate. Flasks were shaken for 16 h to detach other cells, after which, attached primary astrocyte cells were cultured on plates for the performed experiments. Before performing the assays, all cell culture dishes were tested for mycoplasma and were negative.

**RNA preparation and quantitative real-time PCR (qRT-PCR)**

Total RNA was extracted from cells using a NucleSpin RNA kit (ACHEREY-NAGEL, Duren, Germany) according to the manufacturer’s instructions. RNA concentrations were measured using an ND 1000 spectrophotometer (NanoDrop, Wilmington, DE, USA). For cDNA synthesis, 0.8 μg RNA was used with the ReverTra Ace qPCR RT kit (TOYOBO, Osaka, Japan), according to the manufacturer’s instructions. The total reaction volume of the mixture comprised 20 μL with iQ SYBR green supermix (Bio-Rad, Hercules, CA, USA) and primers. Quantification was performed using a LightCycler 480 System (Roche Diagnostics, Almere, the Netherlands), according to the manufacturer’s instructions.

All primers for qRT-PCR were synthesized by Cosmo Genetech (Seoul, South Korea) and primer sequences were as follows:

Mouse *GAPDH* (Forward, 5′-CCATCACCATCTTCCAGGAGCGA-3′; Reverse, 5′-GGATGACCTTGCCCACAGCCTTG-3′),

Mouse *CRTC3* (Forward, 5′-CTTCACAGCACCTGGATGAGAG-3′, Reverse, 5′-TGCTCAGAGCACTCGTGTGAAG-3′),

Mouse *AREG* (Forward, 5′-GCCATTATGCAGCTGCTTTGGACG-3′, Reverse, 5′-TGTTTTTCT TGGGCTTAATCACCT-3′).

Mouse *GFAP* (Forward, 5′-AGAAAGGTTGAATCGCTGGA-3′, Reverse, 5′-CGGCGATAGTCGTTAGCTTC-3′)

Mouse *TUBB3* (Forward, 5′-TGAGGCCTCCTCTCACAAGT-3′, Reverse, 5′-GTCGGGCCTGAATAGGTGTC-3′)

Mouse *Iba1* (Forward, 5′-CAGACTGCCAGCCTAAGACA-3′, Reverse, 5′-AGGAATTGCTTGTTGATCCC-3′)

**Promoter activity**

To screen for *AREG* transcription, whose activation is CRTC3-CREB-dependent, reporter plasmids (pGAS-mAREG-Luc, in which firefly luciferase expression was regulated by 500 base pairs of the 5′ flanking region of the mouse *AREG* promoter) and pRSV-β-gal plasmids were transfected into HEK-293T cells. Twenty-four hours after transfection, the cells were treated with 10 μM FSK for 6 h. Luciferase activity was measured and normalized to β-galactosidase activity. For *CRTC3*-dependent transcription activity screening, cells were co-transfected with *CRTC3,* followed by 6 h incubation with 10 μM FSK. A luciferase assay using an *AREG-CRE* mutant promoter system was also conducted to confirm *AREG-CRTC3-CREB* transcription activity.

**Chromatin immunoprecipitation (ChIP) assay**

C6 rat astrocyte cell line and primary mouse astrocytes isolated from C57BL6/J mice were cultured to 70–80% confluency in T75 flasks and exposed to DMSO or 10 μM forskolin for 45 minutes and then fixed in 1% formaldehyde for 10 min. After fixation, ChIP was performed according to the manufacturer’s instruction (Thermo Scientific) using control IgG (Thermo Scientific) and anti-CRTC3 antibody (Bethyl Laboratories, Inc. Montgomery, TX, USA). The purified ChIP-ed DNA was analyzed using qRT-PCR using the primer sets that amplify the regions spanning the CRE sites located between -91 and -84 bp upstream of the first exon of rat *AREG* genes or CRE sites located between -61 and -54 bp upstream of the first exon of mouse *AREG* genes. For rat: rAREG-Pr-F: 5′-TAATAACCTCCCGCCTCCA-3′ and rAREG-Pr-R 5′-CGAGTCAGGAAGGAGCAGAG-3′ and for mouse: mAREG-Pr-F: 5′-AATCAGGGCTCGATCCCCAC-3′ and mAREG-Pr-R: 5′-TTATAGACCCGCTGGAGCCAC-3′ were used for qRT-PCR analysis.

**Immunohistochemistry**

Mouse brains were fixed with 4% paraformaldehyde (Biosesang, Sungnam, South Korea) for 24 h at 4 °C and washed with PBS. For cryosection, samples were incubated with 30% sucrose in PBS for 24 h at 4°C. The samples were embedded in optimal cutting temperature compound medium (Tissue-Tek, Sakura Finetek USA, Inc. Torrance, CA, USA), snap-frozen on dry ice, and then stored at -80°C until sectioning. Frozen samples were sectioned to 30 μm thickness at -25°C using a Leica CM 1860 (Leica Microsystems, Nussloch, Germany). Free-floating sections were washed with PBS three times for 5 min, blocked with 1% normal goat serum, 0.3% Triton X-100 in PBS for 30 min, and finally incubated with primary antibody against rabbit anti-CRTC3 (ab91654, 1:1,000, Abcam, Cambridge, MA, USA), mouse anti-GFAP (MAB360, 1:1,000, Chemicon-Millipore, Temecula, CA, USA), mouse anti-s100β (ab218515, 1:1,000, Abcam), mouse anti-NeuN (ab104224, 1:1,000, Abcam), goat anti-Iba1 (ab283319, 1:1,000, Abcam), mouse anti-Cre (MAB3120, 1:200, Millipore), rabbit anti-NeuN (#24307S, 1:1,000, Cell signaling), rabbit anti-GFAP (ab7280, 1:1,000. Abcam), or rabbit anti-s100β (GTX129573, 1:200, Genetex) diluted in blocking buffer overnight at 4°C. After washing five times with PBS, a secondary antibody against Alexa Fluor 488 (A-21202, 1:3,000, Invitrogen, Carlsbad, CA, USA), biotinylated anti-rabbit (BA-1100-1.5, 1:3,000, Vector Laboratories, Burlingame, Cam USA), and Alexa Fluor 594 conjugated streptavidin (S11227, 1:3,000, Invitrogen) was added for 60 min at room temperature (RT). Samples were mounted using a fluorescence mounting medium (DAKO, Carpinteria, CA, USA). CRTC3-positive cells were visualized by incubating sections with diaminobenzidine (DAB) solution as the chromogen. For DAB staining, after incubation with primary antibodies, brain tissue sections were incubated with the biotinylated secondary antibody. A VECTASTAIN Elite ABC (Vector Laboratories, Burlingame, CA, USA) reagent mixture was used for 2 h at RT to visualize immunoreactivities. As mentioned above, sections were mounted and covered with coverslip glass.

For Nissl staining, frozen sections on slides are immersed overnight in a 1:1 alcohol/chloroform mix. Afterward, sequential rehydration in 100%, 95% alcohol, and water follows. Tissue sections are stained with 0.1% cresyl violet for 5 minutes, then quickly rinsed. Slides are differentiated in 95% ethyl alcohol (2-30 minutes), assessing staining progress microscopically. Tissue undergoes dual 5-minute dehydration in 100% alcohol, two xylene washes, and final mounting. Protocol aligns with Nissl staining standards.

**Tissue preparation and western blot analysis**

After the fMRI analysis or behavior test, anesthetized mice with isoflurane were perfused with cold PBS. Brains were immediately collected, dissected finely into cortical and hippocampal regions, and stored at -80 ℃ until western blotting was performed. Brain tissues were homogenized with protein extraction buffer (PRO-PREP^TM^, Intron Biotechnology, Korea), and incubated for 30 min on ice. The lysate was centrifuged at 11,000 g for 15 min. An equal amount of protein was separated using SDS-PAGE and then transferred to polyvinylidene difluoride (PVDF) membranes using the transfer apparatus (Bio-Rad Laboratories, Inc., Spain). The membranes were blocked with 0.1% Tween 20 in PBS (PBST) containing 5% fat-free milk powder for 1 h at RT and incubated with primary antibody at 4 °C overnight. The next day, the membrane was washed with PBST and incubated with the conjugated anti-rabbit or mouse IgG-horseradish peroxidase antibodies (Vector Lab, Burlingame, CA, USA) for 1 h at RT. After another wash with PBST, immunoreactive bands were visualized using enhanced chemiluminescent (ECL) substrate (Thermo Fisher Scientific, MA, USA) and detected by exposure to X-ray film. The density of protein bands was analyzed using Image J software (NIH, Bethesda, MA, USA) and normalized to loading controls. The following primary antibodies were used for western blot analysis: rabbit anti-CRTC3 (#2720, 1:1,000), mouse anti-phospho-CREB(ser133) (#9198, 1:1,000), mouse anti-CREB (#9104, 1:1,000) (Cell Signaling Technology, Danvers, USA), mouse anti-AREG (sc-74501, 1:500, Santa Cruz Biotechnology, CA, USA), rabbit anti-phopho-GluA1(Ser831) (#04-823, 1:1,000, Millipore), and rabbit anti-GluA1 (#AB1504, 1:1,000, Millipore). For loading controls, immunoblotting for mouse anti-β-actin (A5441, 1:5,000) was purchased from Sigma-Aldrich.

**Genotyping**

Genomic DNA was prepared from tail biopsies and genotyped using the following primer sets. For systemic CRTC3 null mice: *CRTC3* WT allele, 5′-CCTGAGTTATTGGCGGATGT-3′ and 5′-CACTCAGGCTGTAGCA-AGCA-3′; CRTC3 KO allele, 5′-ATGGAAGGATTGGAGCTACG-3′ and 5′-CACTCAGGCTGTAGCAAGCA-3′. For CRTC3 floxed mice: 5′-AAACTAACTGGGCCTGAGTCCATCC-3′, 5′-CAGGTGCTGTGAAGAATAACTGGC-3′, and 5′-TTCATGGATGCCACTCTCAGAAGCC-3′

**Behavioral tests**

The behavioral tests were performed within one month before infusion and within the third week of drug infusion. Animals were kept in the testing room 30 minutes before the test to allow habituation to the new environment.

1. **Tube dominance test and video analysis**

Social superiority was measured using the tube dominance test. C57BL/6 mice were housed in groups of 2 or 3 for at least four weeks before the test. CRTC KO or CRTC3 fl/fl mice were housed in groups three weeks after being separated from their mother. The tube test apparatus is a 30 cm long transparent plastic tube with a 2.5 cm inner diameter. The diameter size permits an adult mouse to move through the tube without reversing direction. Before the tube dominance test trials, all mice were adapted to the tube by guiding them through it from both ends for 2 consecutive days. During the training period, the mouse entered the tube as naturally as possible without undue stress. After training, each mouse was introduced to opposing ends of the tube and the two mice meet in the middle. When one mouse pushed the opponent mouse out of the tube, it scored 1 point, and this test was repeated three times. If the two mice did not meet in the middle of the tube, the experiment was repeated. The experiment did not exceed 2 min; if it exceeded 2 min, the experiment was repeated. For example, if one mouse scores 2 or 3 victory points, it wins, and the other mouse loses. The ranking system was conducted as follows, based on an experiment using three mice from a housing cage. Tube tests were conducted following round-robin design to randomize the test order (1, 2 and 3 are mouse naming): 1 vs 2, 1 vs 3 and 2 vs 3. If mouse 1 wins twice, it is ranked first. If mouse number 2 loses to mouse number 1 and wins against mouse number 3, then it is ranked second. Mouse number 3, having lost to both mice, is ranked third. For each test, the winning was determined by the number of winning points. The tube was sterilized with 70% ethanol for each trial.

Video analysis allows the push to be identified when one mouse slides its head under the other. The tube test behavior was identified from the meeting point to the end of the tube test trial. Push-initiation is when a mouse pushes the opponent. Push-back is defined as a counter-push after being pushed by the opponent and retreat indicates back-out when the opponent pushed or voluntarily withdraws. Stillness shows no movement and resistance meant the behavior of holding on to the territory when being pushed. Resistance and retreat results analysis were excluded if the loser mouse was immediately pushed from the center to the end of the tube.

1. **Forced swimming test**

Briefly, mice were made to swim in a glass cylinder (19 cm high × 15 cm in diameter) filled with water (24 ± 1 °C) up to 15 cm in depth. This procedure was performed over two consecutive days. In the pre-test session, each mouse was individually subjected to forced swimming for 15 min. On the second day, the same mouse was forced to swim for 5 min. Each mouse was considered immobile when it tried struggling and then floated motionless in the water. The duration of swimming, struggling, and immobility was recorded. The glass cylinder was cleaned between tests, and the water was replaced.

1. **Y-maze test**

The Y-maze test involved a three-arm horizontal maze (10 × 50 × 20 cm) in which the arms were symmetrically separated at 120°. The short-term memory test was conducted as follows: in the first session, one arm of the maze was hidden, and two arms were opened. A mouse was placed at the center of the maze and could freely explore the two open arms for 10 min. The test session was conducted 3 h later; the closed arm was opened to allow mice to explore. A mouse was placed at the center of the maze and could freely explore the three open arms for 5 min. The test was recorded and measured using the SMART video-tracking system (software version 3.0, Panlab, Harvard Apparatus, Barcelona, Spain). The data were analyzed for the time and number of entries in the newly opened arm. The maze was cleaned with 70% ethanol between tests to remove residual odor.

1. **Morris water maze test**

The water maze was performed with a circular pool (50 cm high × 120 cm diameter, water at 24 ± 1 °C). For the experiment, a circular platform (10 cm diameter) was located 1.5 cm beneath the water surface in the center of the NW quadrant. The pool was filled to a depth of 40 cm with water containing skim milk powder, which was replaced each day. Extra maze cues comprised geometric shapes on the walls, posters, and furniture located in the room in which the water maze was situated. One cue was attached to the side of the water maze pool near the platform.

The experimental procedure comprised 5 days of testing, with 4 trial days and a probe test day. During trial days, a mouse was placed in one of the three quadrants facing the wall and allowed to find the escape platform for 1 min. If it found the platform within the given time, it remained there for 1 min; otherwise, it was guided to the platform and stayed there for 1 min. Each mouse was given three training sessions, and 1 min of rest was provided between each training session. In the three training sessions, the mouse was placed in different quadrant positions (NE, SW, and EW) without locating the platform zone. After the training days, a probe test was conducted without a platform. The mouse was placed in the SE quadrant facing the wall and allowed to find the platform. Every experiment was recorded and measured using the SMART video-tracking system (software version 3.0, Panlab, Harvard Apparatus). The data were analyzed for the swimming time (in s) and swimming distance (in cm) needed to reach the platform.

1. **Elevated plus-arm maze**

The elevated plus-arm maze apparatus comprised a plus-shaped maze, with two open (10 × 50 cm) and two closed (10 × 50 × 40 cm) arms, elevated to 60 cm above ground level. A mouse was placed at the center of the apparatus and could explore the maze freely for 5 min. The test was recorded and measured by the SMART video-tracking system (software version 3.0, Panlab, Harvard Apparatus). The data were analyzed for the time taken to enter and the number of entries into the open and closed arms. The maze was cleaned with 70% ethanol between tasks to remove residual odor.

1. **Tail suspension test**

Mice were suspended above the ground with tape attached to its tail. The distance between the mice to the ground was 30 cm for each trial. Mouse behavior was recorded on video for 6 min, and total immobility time was analyzed manually.

1. **Strength and grid tests**

For the strength test, mice were allowed to grasp a steel scrubber, which was attached to a weight (35 g). Then, the mice were raised by their tail. The time until the mice dropped the weight was measured. This procedure was performed over 3 consecutive days.

For the grid test, mice were allowed to grasp onto a steel wire mesh (15 × 20 cm) on the ground. Then, the mesh was flipped and the time until the mouse let go of the mesh was measured. This procedure was performed over 2 consecutive days.

1. **Olfactory preference test**

The olfactory test is a sensory assay used to measure the olfactory ability to sense attractive or aversive scents. Flavors were prepared by soaking filter paper (2 × 2 cm) with 10% peanut butter diluted in water for the preference behavior and 10% 2-methyl butyric actin (2-MA) diluted in water for the avoidance behavior. Four clean cages were prepared without bedding and lined up in the experimental lab. The mice were allowed to explore the three cages sequentially for 15 min. Between each cage, the next cage was blocked with a large piece of filter paper to keep it from being seen. After 15 min of habituation in the fourth cage, the filter paper with diluted peanut butter was placed on the opposite side of the mouse for 3 min. The inspecting times were measured and recorded. After a 1 min break, the test trial was repeated with 2-MA scent paper into the corners of the opposite side. Mice’s behavior was recorded on a video camera to measure the time spent for the mouse’s nose to be 1 mm from the filter paper. This test cage was cleaned with two tissue papers sprayed with 70% ethanol.

1. **Urine scent marking test**

Mice were placed in a compartment of a clean cage without bedding divided by a meta grid. A smooth barrier was placed on top of the cage to prevent escapes. A sheet of filter paper the same size as the bottom of the cage was placed on each cage to collect urine. Each pair of mice was placed on opposite sides of a separated partition section for 2 h. The marked paper was stained with Ninhydrin spray reagent (Sigma-Aldrich) and scored using a 1 cm^2^ grid overlay. All squares with a scent mark were counted. The urine mark size delineates territorial boundaries and shows social status. The winner of each pair possessed a higher proportion of squares of urine marks.

1. **Novel object recognition test**

Mice were placed in a rectangular box (30 × 30 × 30 cm) without objects for 15 min for habituation to the experimental environment. In the second session, two identical objects were placed into both sides of the cages and 5 cm away from the walls. Mice were allowed to explore the objects for 5 min and then were placed back into their home cages for 4 h. In the third session, one object was replaced with a novel object with a different color and shape. Mice were placed again in the testing cage and allowed to explore the familiar and the novel objects for 5 min. The test was recorded and measured using the SMART video-tracking system (software version 3.0, Panlab, Harvard Apparatus). The data were analyzed for the exploration time of the two objects. The maze was cleaned with 70% ethanol between tasks to remove residual odor.

**Affymetrix whole transcript expression arrays**

Affymetrix whole transcript expression arrays were conducted according to the manufacturer's protocol (GeneChip whole transcript PLUS reagent kit, Thermo Scientific, Rockford, IL, USA). cDNA was synthesized using a GeneChip whole transcript amplification kit as described by the manufacturer. The sense cDNA was then fragmented and biotin-labeled with terminal deoxynucleotidyl transferase using a GeneChip whole transcript terminal labeling kit. Approximately 5.5 μg of labeled DNA target was hybridized to an Affymetrix GeneChip mouse 2.0 ST array at 45 °C for 16 h. Hybridized arrays were washed and stained on a GeneChip fluidics station 450 and scanned on a GCS3000 scanner (Affymetrix, Santa Clara, CA, USA). Signal values were computed using the Affymetrix GeneChip Command Console software (AGCC).

**Raw data extractions and statistical analyses of microarray data**

Raw data were extracted automatically with the Affymetrix data extraction protocol using the software provided by AGCC. After importing CEL files, the data were summarized and normalized using a robust multi-average (RMA) method implemented in the Affymetrix Expression Console software. We exported the results obtained using gene-level RMA analysis and performed differentially expressed gene (DEG) analysis.

The comparative analysis between the test and control sample was conducted using fold-change. For a DEG set, hierarchical cluster analysis was performed using complete linkage and Euclidean distance as a measure of similarity. To obtain a list of significant probes, gene-enrichment and functional annotation analysis was performed using gene ontology (http://geneontology.org/) and Kyoto Encyclopedia of Genes and Genomes (KEGG) (http://kegg.jp).

All statistical tests and visualization of DEGs were conducted using R statistical language version 3.1.2. (www.r-project.org).

**Supplementary Figures**

**
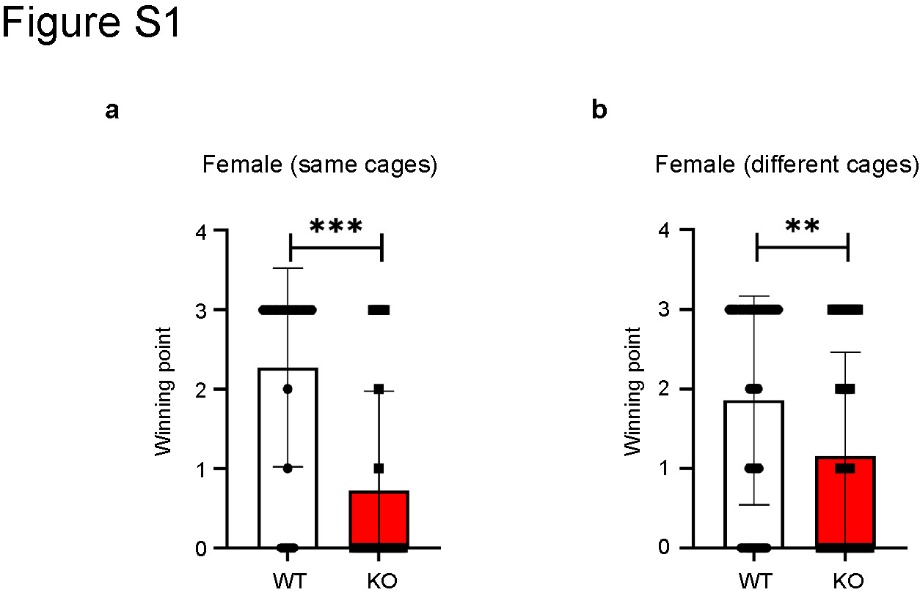
**

**Supplementary Figure 1:** CRTC3 KO female mice show lower social dominance rank.

**a,** Number of winning points in the same cages, WT (n = 19), KO (n = 19). b, Number of winning points in different cages, WT (n = 70), KO (n = 70). Mann-Whitney test, **p < 0.01, ***p < 0.001, Error bars, SEM.

**
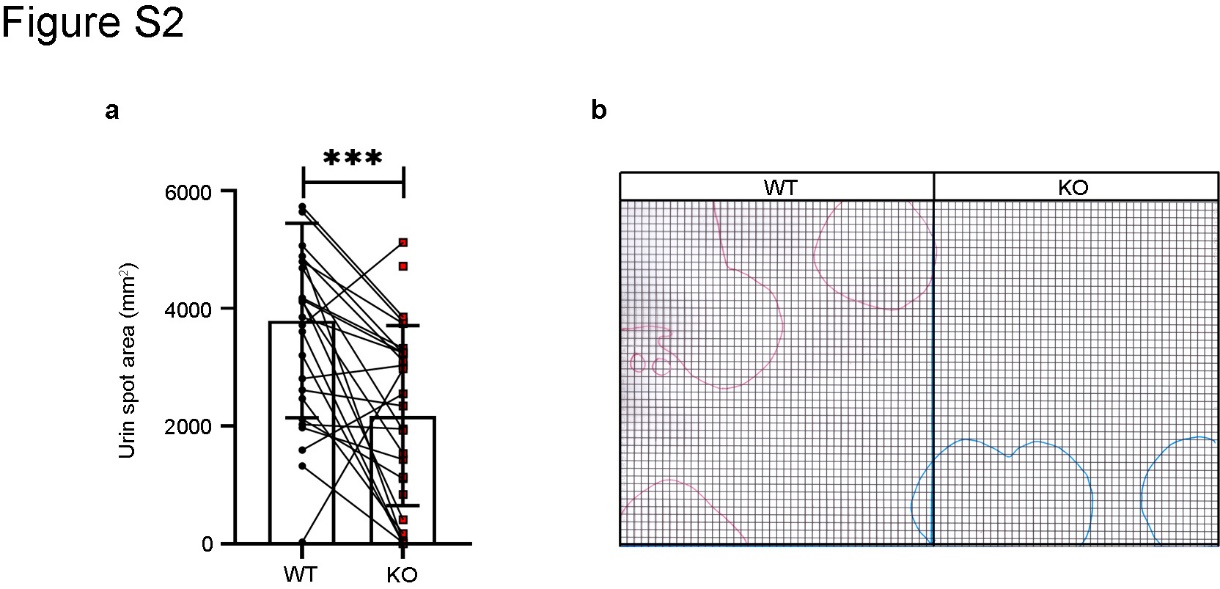
**

**Supplementary Figure 2:** CRTC3 KO mice had a lower urine spot area.

**a,** Quantification of the urine area. WT (n = 27), KO (n = 27). **b,** A filter paper was spotted with the urine of the wild-type mice (WT) and CRTC3 KO mice (KO), Wilcoxon signed test, ***p < 0.001. Error bars, SEM.


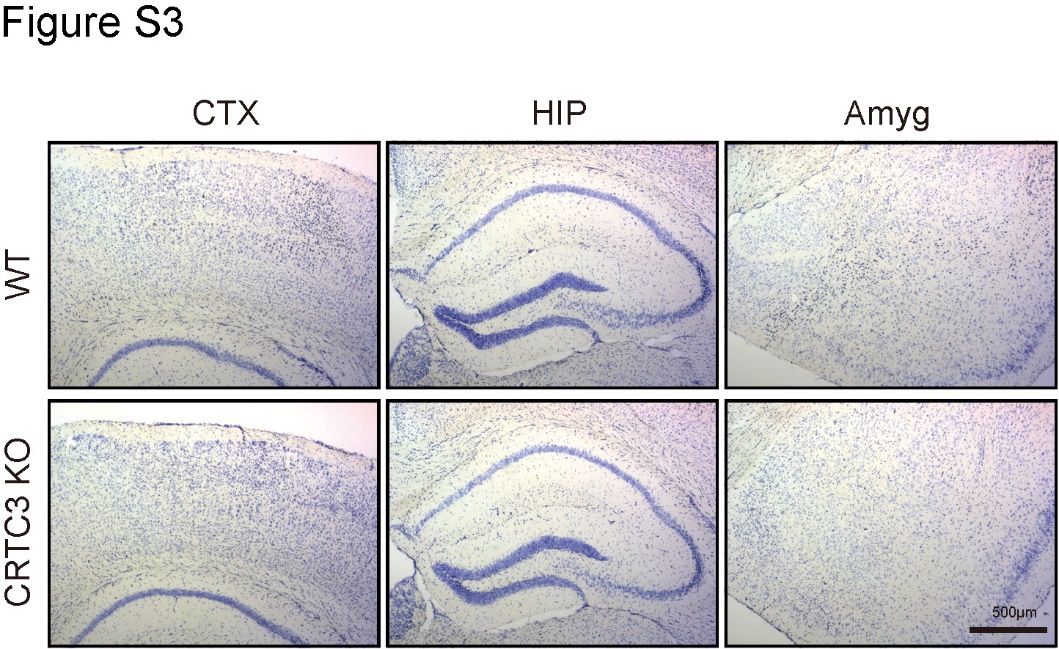


**Supplementary Figure 3:** Nissl-stained mouse brain sections in WT and CRTC3 KO mice. WT n = 3, CRTC3 KO n = 3. Scale bar: 500 μm


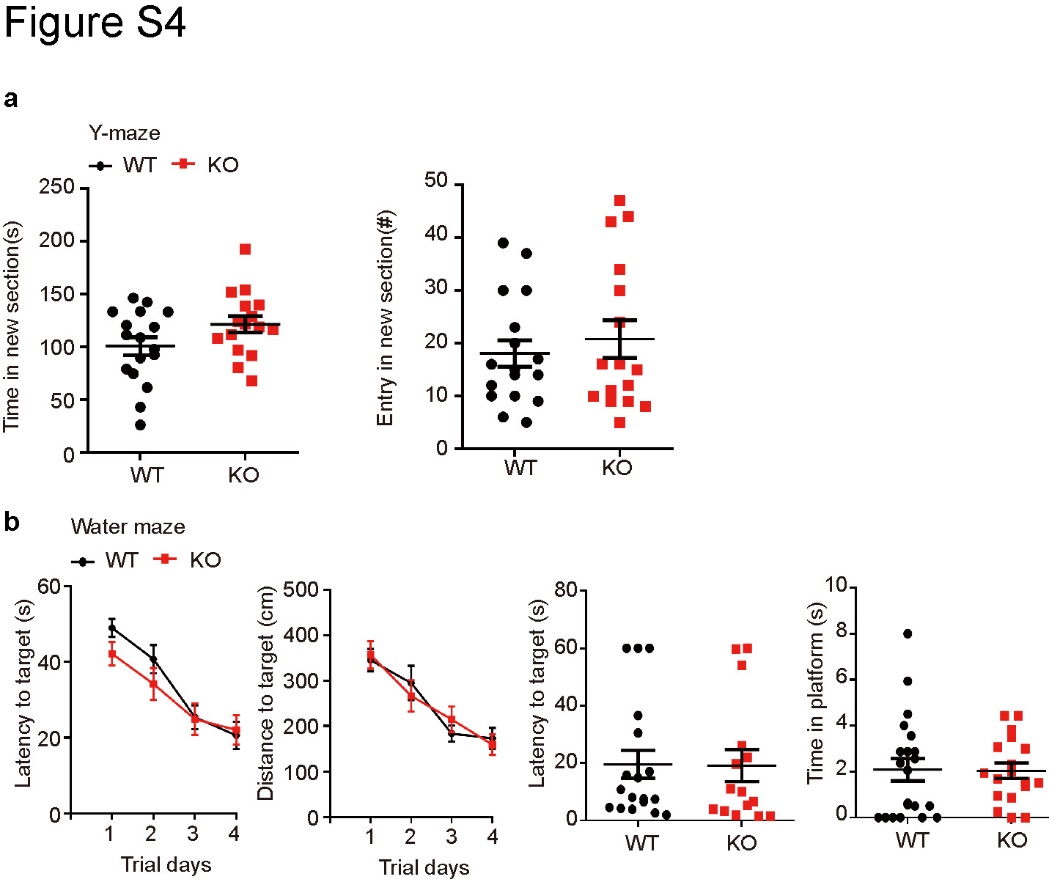


**Supplementary Figure 4:** WT and CRTC3 KO mice do not show differences in memory formation.

**a,** The time spent (left) and number of entries (right) in the new section of the Y-maze test by WT (black) and CRTC3 KO (red) mice, WT (n = 17), KO (n = 16). **b,** The latency time to reach the target platform and distance to the platform on the trial days (left), and the latency time to reach the platform and time spent on the platform on the test day (right) in the water maze test by WT (black) and CRTC3 KO (red) mice, WT (n = 21), KO (n = 18). Unpaired t-test, ANOVA test (Bonferroni’s multiple test), p = ns, Error bars, SEM.


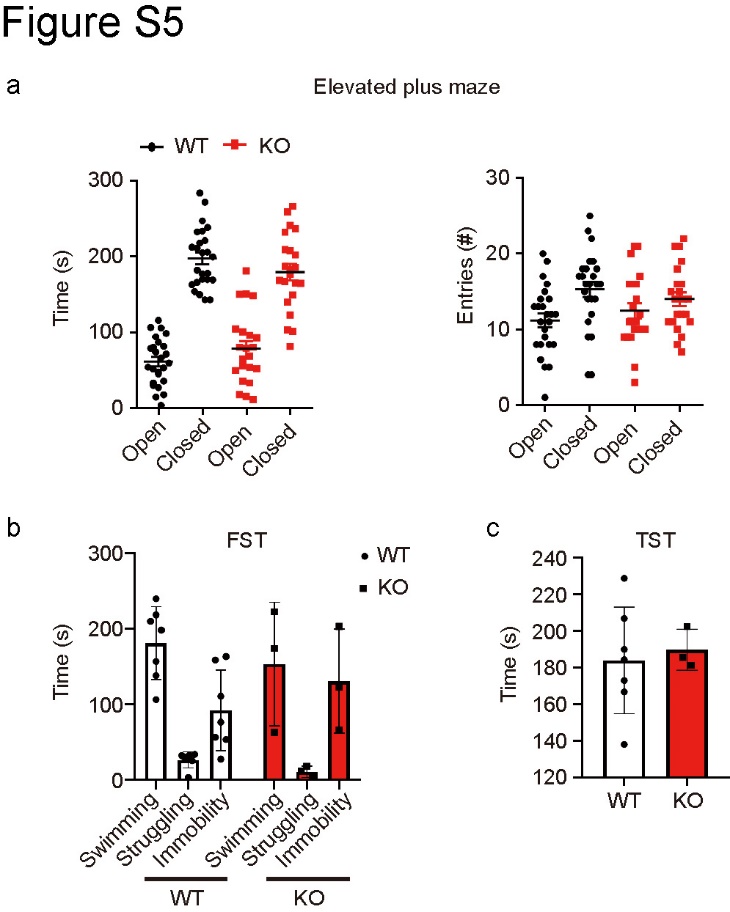


**Supplementary Figure 5:** WT and CRTC3 KO mice do not show differences in mood.

**a,** Time spent in each arm (left) and the number of entries into each arm made by WT and CRTC3 KO mice in the elevated plus-arm maze (EPM), WT (n = 25), KO (n = 22). **b,** The number of times behavior phenotypes are displayed in the forced swim test (FST) in WT and CRTC3 KO mice. WT (n = 36), KO (n = 24). **c,** The total immobility time in the tail suspension test (TST), WT (n = 34), KO (n = 21), One-way ANOVA test, Unpaired t-test. Error bars, SEM.


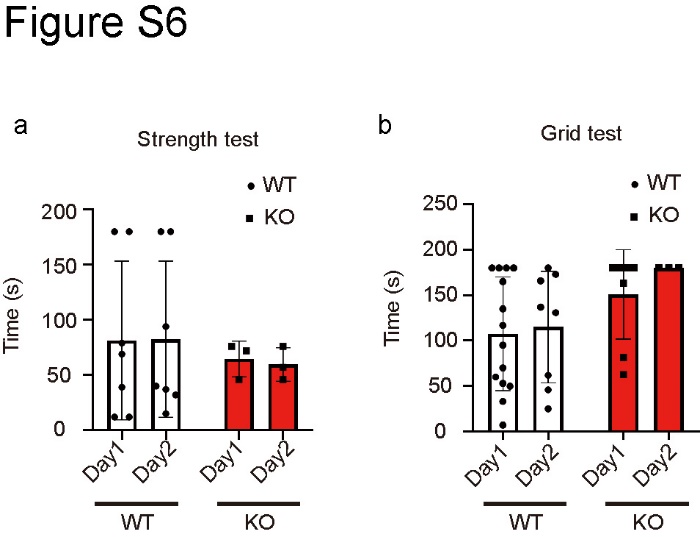


**Supplementary Figure 6:** WT and CRTC3 KO mice do not have differences in strength.

**a,** The time holding weights in the strength test. WT (n = 7), KO (n = 3). **b,** the time holding the grid in the grid test. WT (n = 14), KO (n = 8). ANOVA test (Tukey’s multiple test), p = ns, Error bars, SEM.


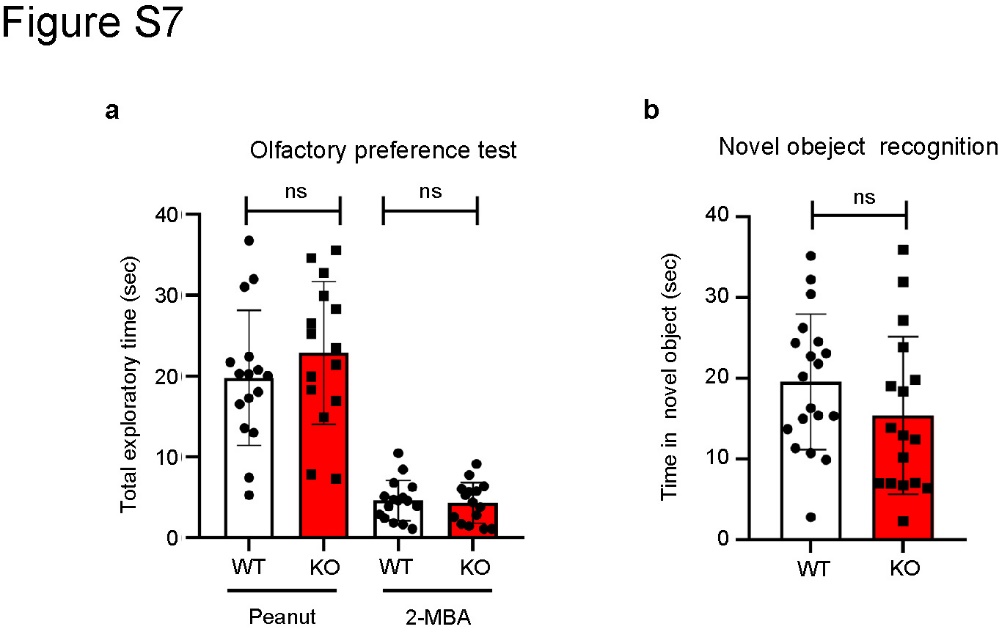


**Supplementary Figure 7:** WT and CRTC3 KO mice do not have differences in olfactory preference tests and novel object recognition.

**a,** olfactory preference test WT (n = 16), KO (n = 15). **b,** Novel object recognition WT (n = 19), KO (n = 17). Unpaired t-test, p = ns, Error bars, SEM.


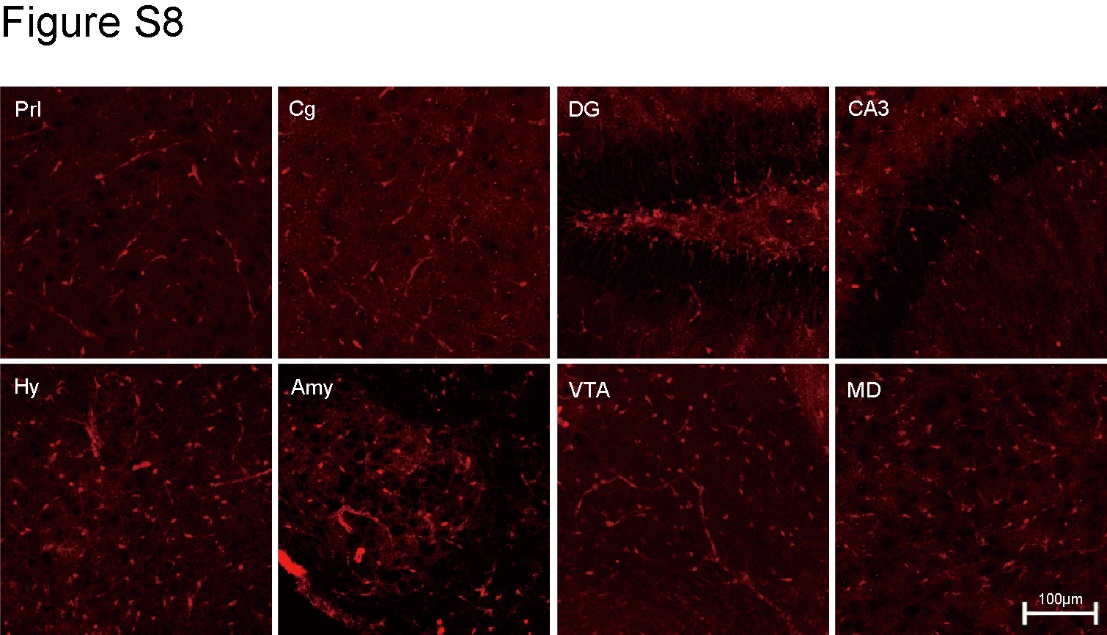


**Supplementary Figure 8: CRTC3 is widely expressed in several areas in the mouse brain.** Representative immunohistochemistry images showing several brain regions stained using an antibody against CRTC3. Prl, prelimic cortex; Cg, cingulate gyrus; DG, dentate gyrus; CA3, cornu ammonis 3; Hy, hypothalamus; Amy, amygdala; VTA, ventral tegmental area; MD, mediodorsal thalamus


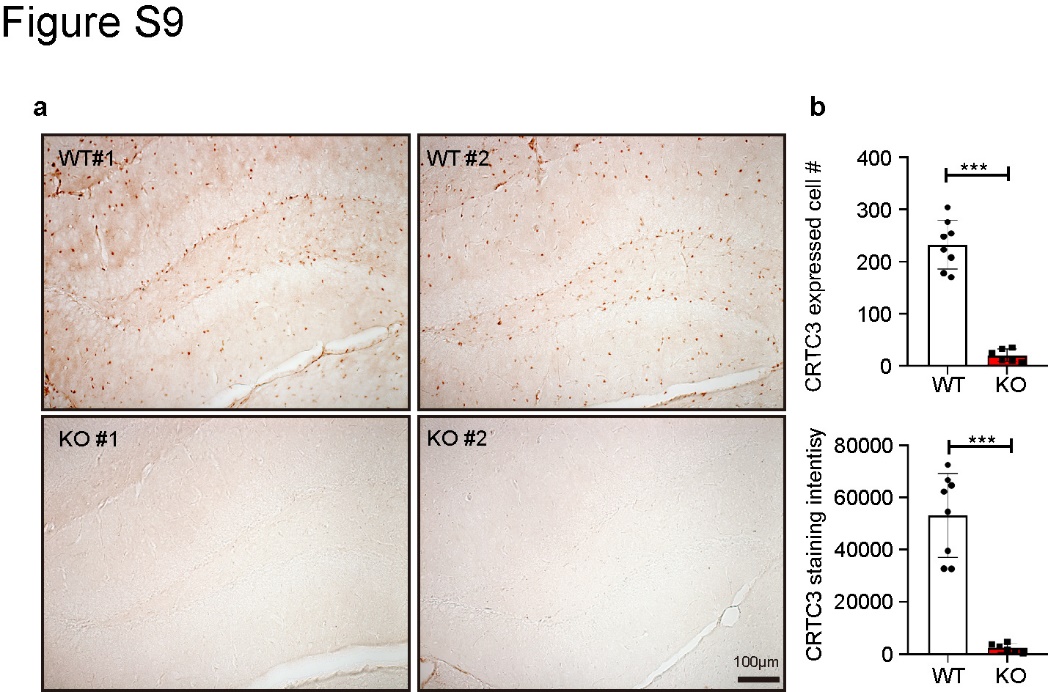


**Supplementary Figure 9:** CRTC3 KO mice do not express CRTC3 in the brain.

**a,** Representative immunohistochemistry images showing CRTC3 staining in wild-type (WT) and CRTC3 KO (KO) mice. **b,** The number and intensity of CRTC3 staining in the mouse brain. WT (n = 8), KO (n = 7). Mann-Whitney test, ***p < 0.0001, Error bars, SEM.


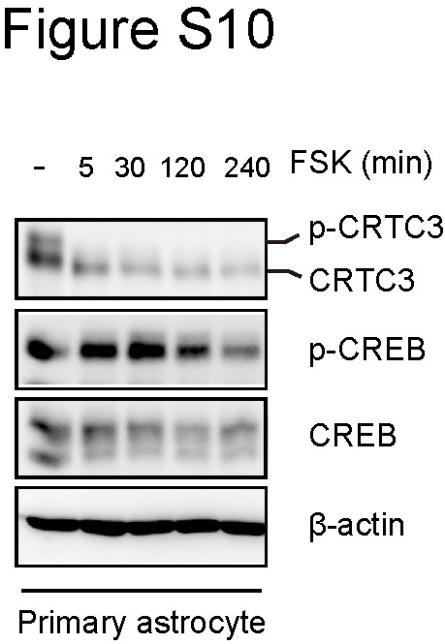


**Supplementary Figure 10**: Immunoblot assay showing the effect of forskolin (FSK) with different exposure times on CRTC3 de-phosphorylation and CREB phosphorylation in primary astrocytes.


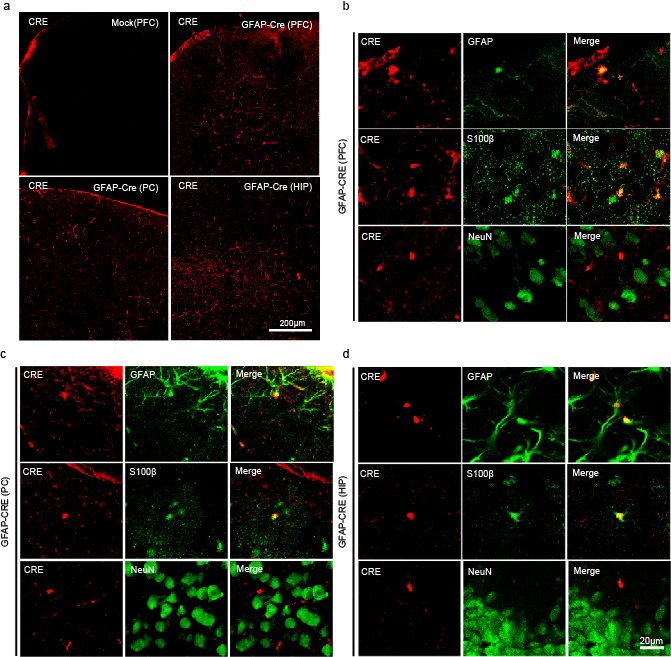


**Supplementary Figure 11: a,** Representative immunofluorescence images of Cre expression (Red) in the prefrontal cortex (PFC), parietal cortex (PC), and hippocampus (HIP) of CRTC3-fl/fl mice at 14 days following administration of AAV5 virus encoding GFAP-Cre. Scale bar: 200 μm **b,** Representative immunofluorescence images of Cre (Red) with GFAP (astrocyte, Green), S100β (astrocyte, Green) or NeuN (neuron, Green) in PFC of CRTC3fl/fl mice at 14 days following administration of AAV5 virus encoding GFAP-Cre. **c,** Representative immunofluorescence images of Cre (Red) with GFAP (astrocyte, Green), S100β (astrocyte, Green) or NeuN (neuron, Green) in PC of CRTC3fl/fl mice at 14 days following administration of AAV5 virus encoding GFAP-Cre. **d,** Representative immunofluorescence images of Cre (Red) with GFAP (astrocyte, Green), S100β (astrocyte, Green) or NeuN (neuron, Green) in HIP of CRTC3fl/fl mice at 14 days following administration of AAV5 virus encoding GFAP-Cre. Scale bar: 20 μm for b, c, and d


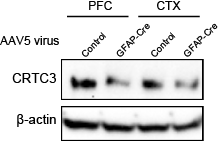


**Supplementary Figure 12:** Immunoblot assay showing the downregulation of CRTC3 in the prefrontal cortex PFC and cortex (CTX) region after administration of GFAP-Cre virus into CRTC3 fl/fl mice.

**
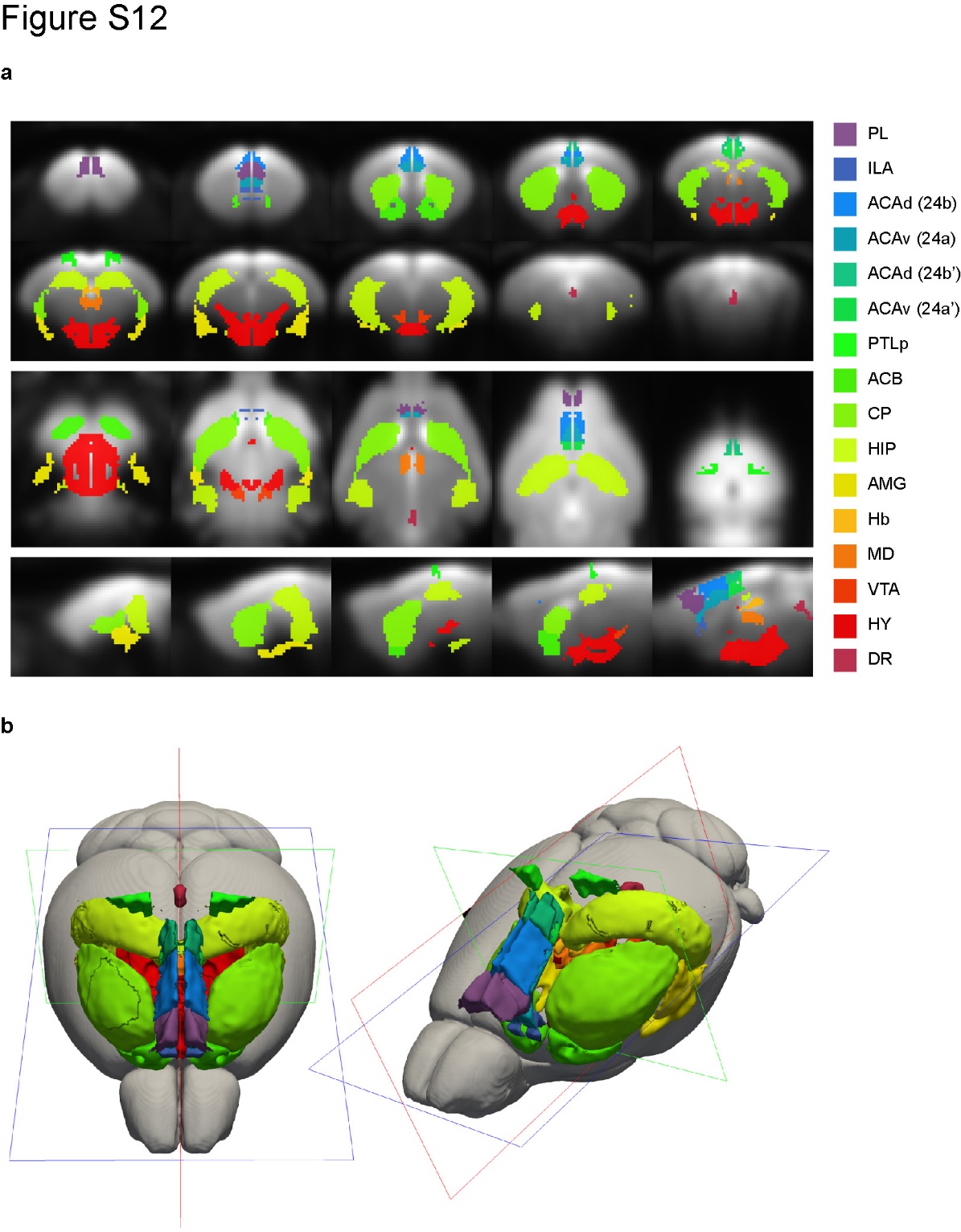
**

**Supplementary Figure 13:** Regions-of-interest (ROIs) definitions of mouse social dominance-related brain areas.

**a,** Spatial maps of 31 ROIs are displayed in different colors overlaid on averaged EPI data. **b,** 3D-reconstructed maps of ROIs are represented.


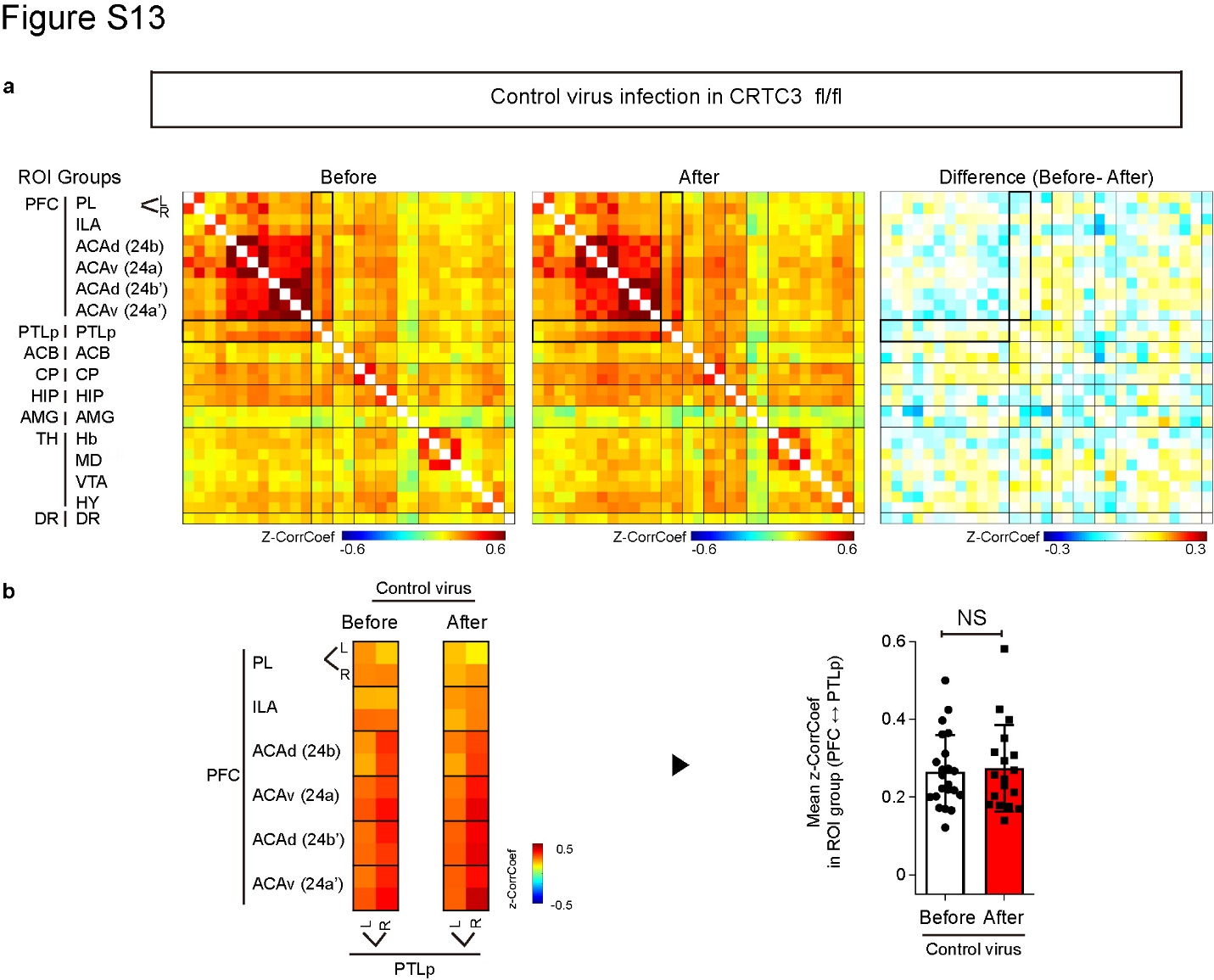


**Supplementary Figure 14:** Resting-state correlation matrix for all pairs of seed ROIs related to the social dominance behavior in control AAV-infected CRTC3 fl/fl mice mice.

**a,** The resting-state functional connectivity matrices of social dominance-related brain ROIs from control adeno-associated virus (AAV) in pre-injection states (left) and in post-injection states (middle) are represented. The mean difference map is represented (right). **b,** Compared with the post-injection state, Control AAV-infected CRTC3 fl/fl mice also show no significant difference for the mean z-CorrCoef across all focused matrices between the ROI groups of the prefrontal cortex (PFC) and posterior parietal cortex (PTLp). L: left and, R: right hemispheres. before (n = 22), after (n = 18), Unpaired t-test, p = ns. Error bars, SEM.


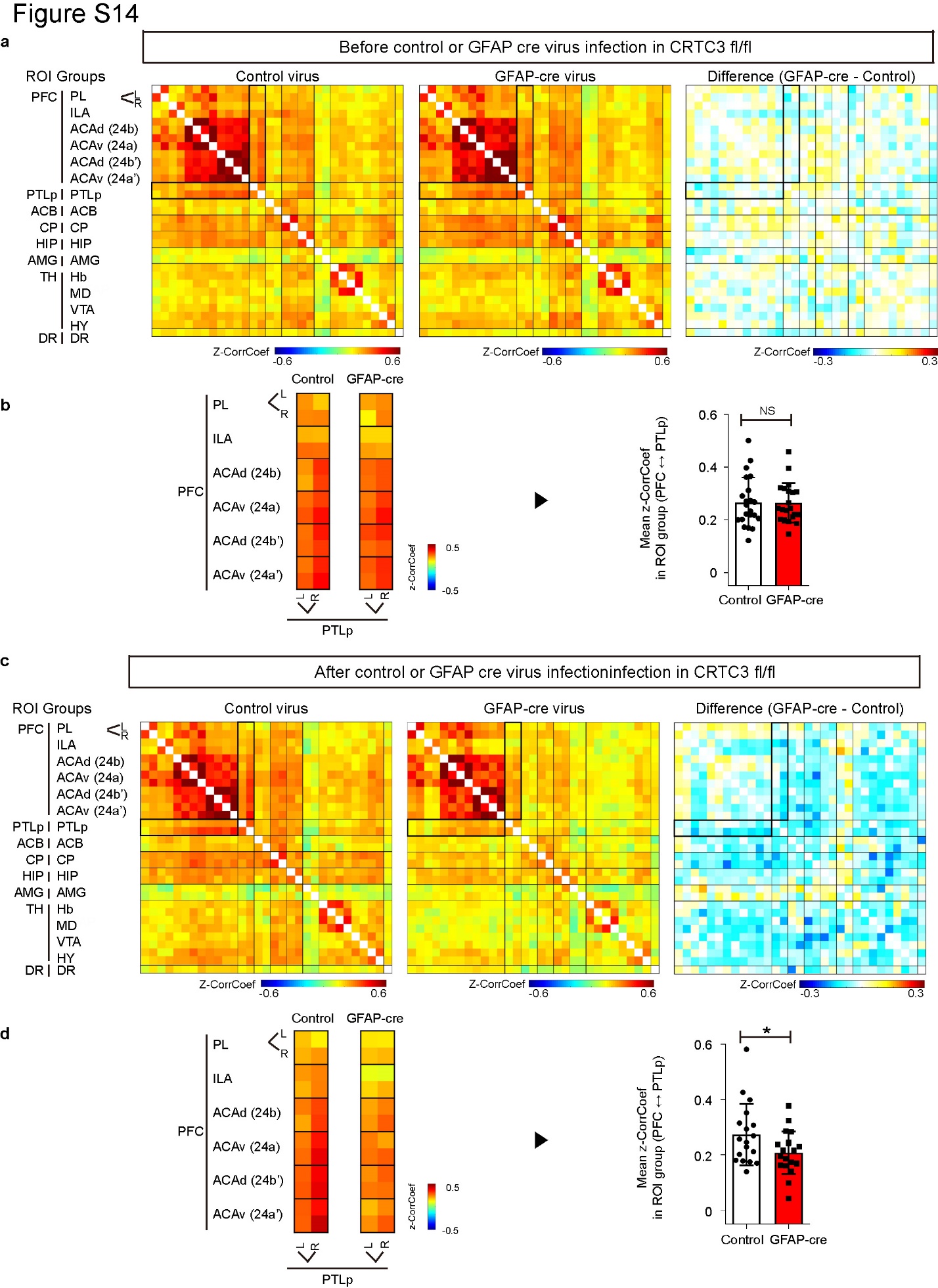


**Supplementary Figure 15:** Resting-state correlation matrix for all pairs of seed ROIs related to the social dominance behavior in astrocyte-specific CRTC3-knockout (GFAP-Cre) mice.

**a,** The resting-state functional connectivity matrices of social dominance-related brain ROIs from control (left) and GFAP-Cre adeno-associated virus (GFAP-Cre, middle) pre-injection states are represented. The mean difference map is represented (right). **b,** Compared with the control AAV injection, GFAP AAV-infected CRTC3 fl/fl mice also show no significant difference for the mean z-CorrCoef across all focused matrices between the ROI groups of the prefrontal cortex (PFC) and posterior parietal cortex (PTLp). L: left and, R: right hemispheres. Control (n = 22), GFAP-cre (n = 21). **c,** The resting-state functional connectivity matrices of social dominance-related brain ROIs from control (left) and GFAP-Cre (middle) post-injection states are represented. The mean difference map is represented (right). **d,** Compared with the control, GFAP-Cre AAV-infected CRTC3 fl/fl mice show significantly decreased mean z-CorrCoef across all focused matrices between the ROI groups of the prefrontal cortex (PFC) and posterior parietal cortex (PTLp). control (n = 18), GFAP-Cre (n = 20), Unpaired t-test, p = ns. *p < 0.05. Error bars, SEM.

**
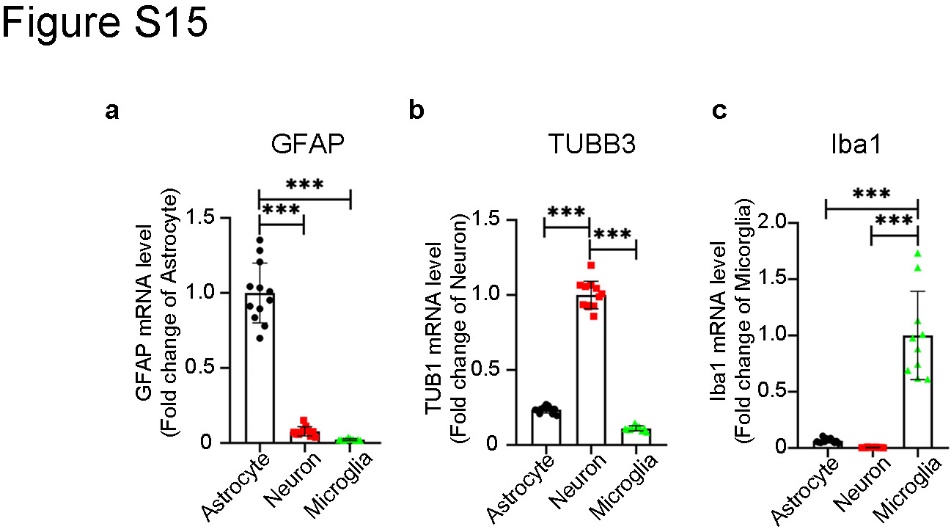
**

**Supplementary Figure 16: Relative cell marker mRNA expression in primary cell cultures. a,** *GFAP* marker for astrocytes. **b,** *TUBB3* (βIII-tubulin) maker for neurons. **c,** *Iba1* marker for microglia. Astrocytes (n = 12), neurons (n = 12), microglia (n = 10), Unpaired t-test. ***p < 0.001. Error bars, SEM.


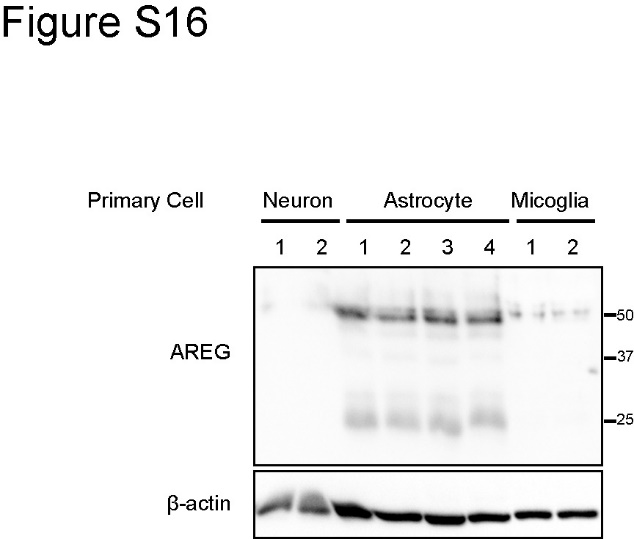


**Supplementary Figure 17:** Immunoblot assay showing the specific expression of amphiregulin in astrocytes.


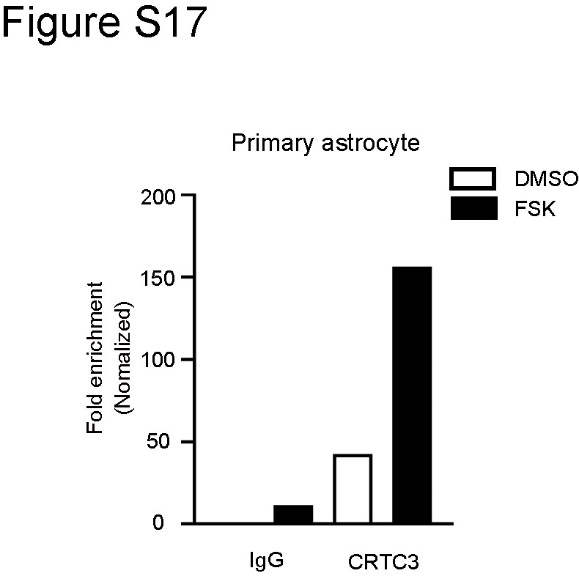


**Supplementary Figure 18:** Chromatin immunoprecipitation (ChIP) assay showing occupancy of CRTC3 over the AREG promoter in primary mouse astrocytes exposed to forskolin (FSK) as indicated. Data are presented as the mean ± SEM and statistical significance was determined by an unpaired Student's t-test using GraphPad Prism 5 software.


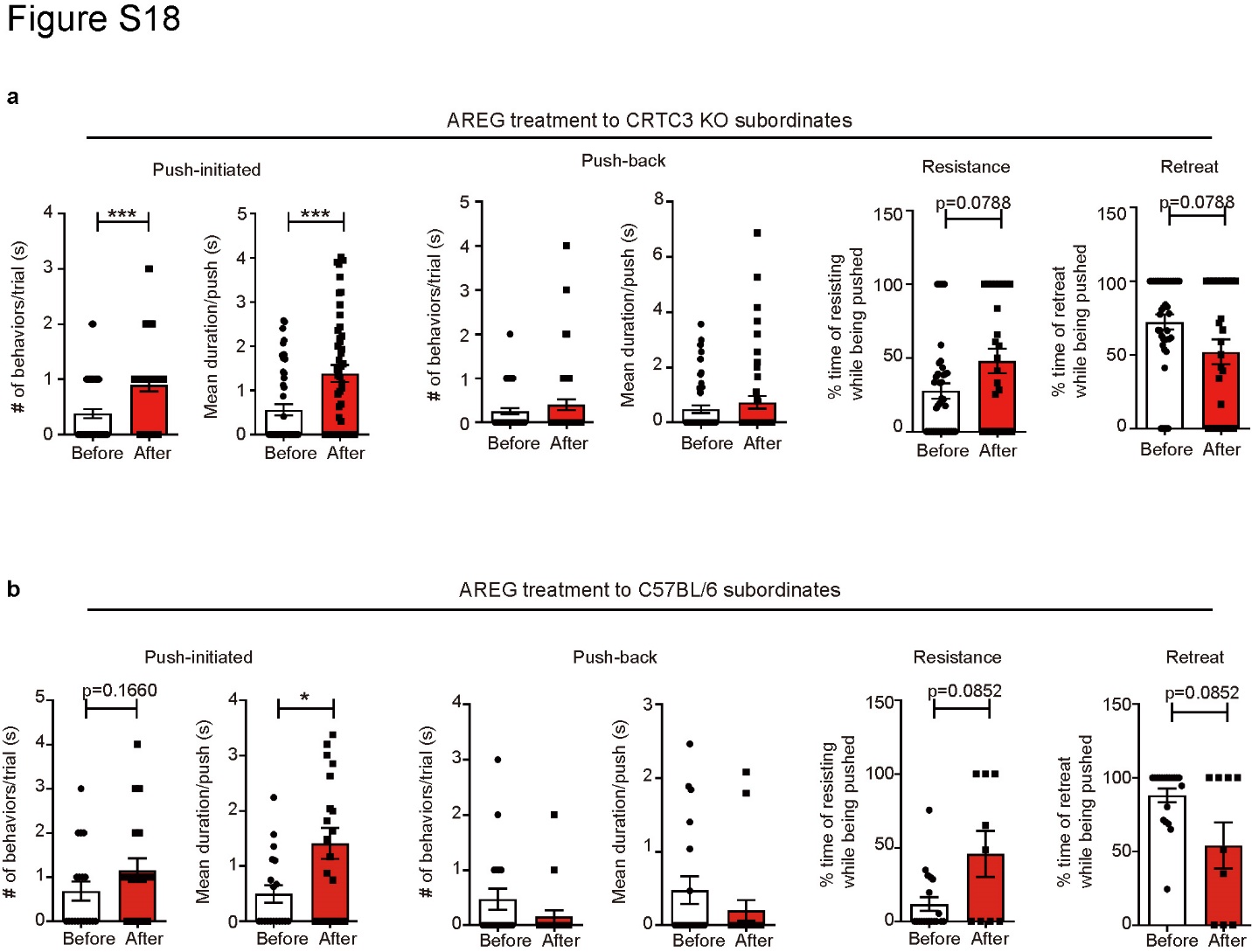


**Supplementary Figure 19**: AREG treatment of subordinates increases push-initiation and resistance.

**a,** Changes in behavioral displays in CRTC3 KO subordinates treated with AREG in the tube dominance test. Push-initiated, Push-back; Before (n = 47), after (n = 47), Resistance, Retreat; Before (n = 40), after (n = 26). **b,** Changes in behavioral displays in C57BL/6 subordinates treated with AREG in the tube dominance test. Push-initiated, Push-back; Before (n = 19), after (n = 19), Mann-Whitney test, ***p < 0.0001, p = ns, Error bars, SEM.


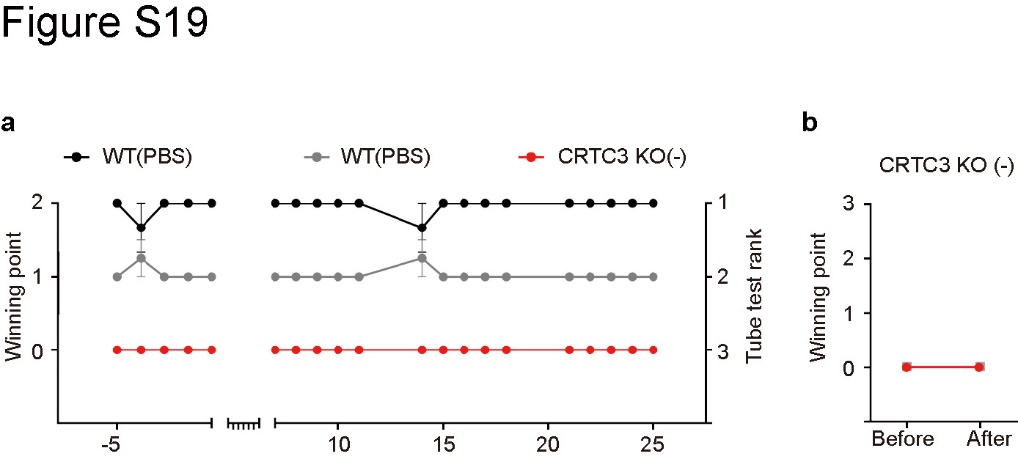


**Supplementary Figure 20:** No change in the tube rank in cages with vehicle-treated CRTC3 KO mice.

**a,** Summary changes of the tube dominance test in vehicle-treated CRTC3 KO mice. **b,** The average number of winning points scored (red) before and after AREG infusion in CRTC3 KO mice.


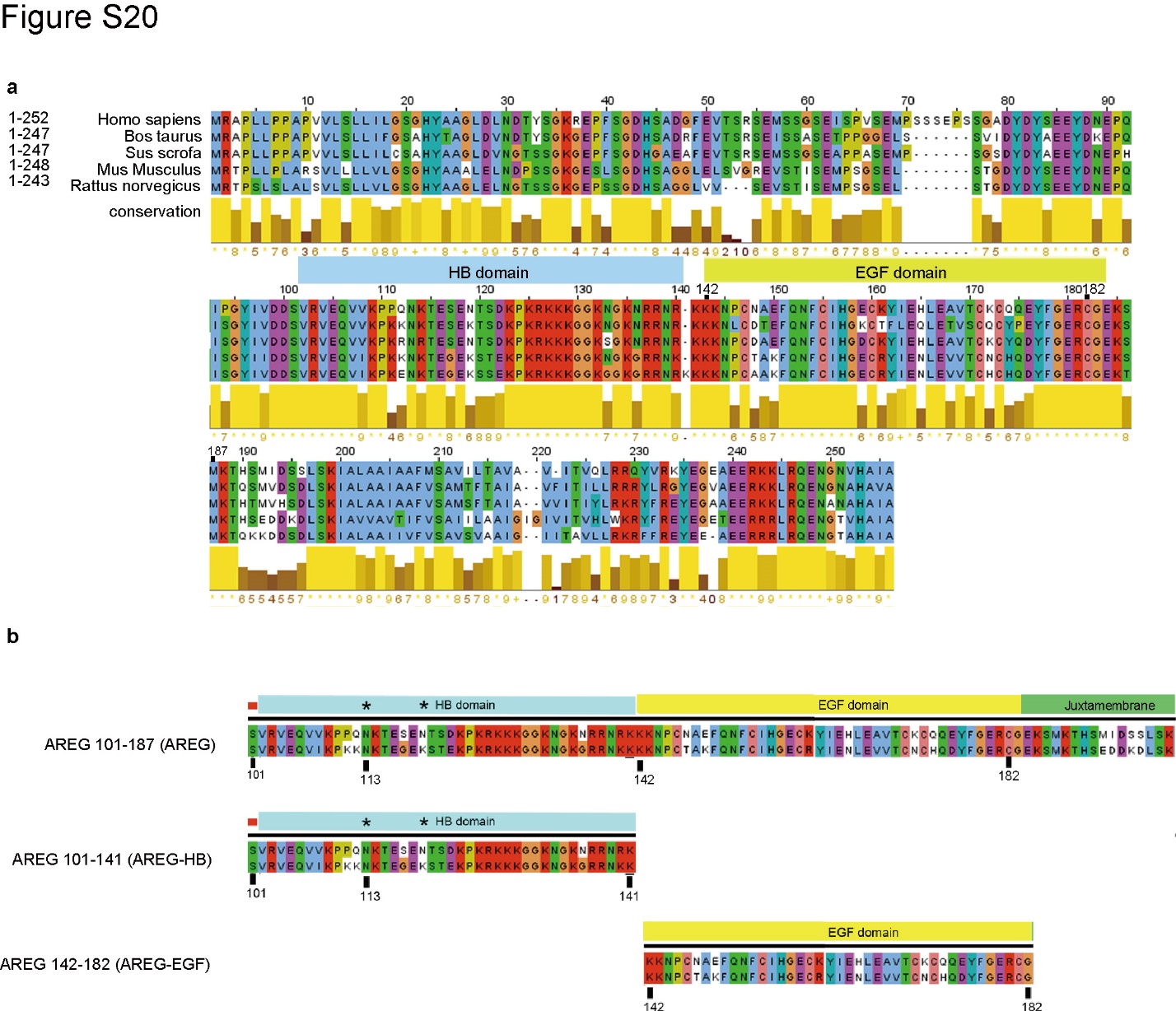


**Supplementary Figure 21:** Epidermal growth factor (EGF) and heparin-binding (HB) domains of AREG are conserved among several species.

**a,** Alignment of the AREG protein sequence among various species, *Homo sapiens* (human), *Bos Taurus* (cattle), *Sus* *scrofa* (common wild pig), *Mus musculus* (mouse), *Rattus norvegicus* (rat). **b,** Selected sequence that was synthesized as AREG-HB and AREG-EGF domain peptides.


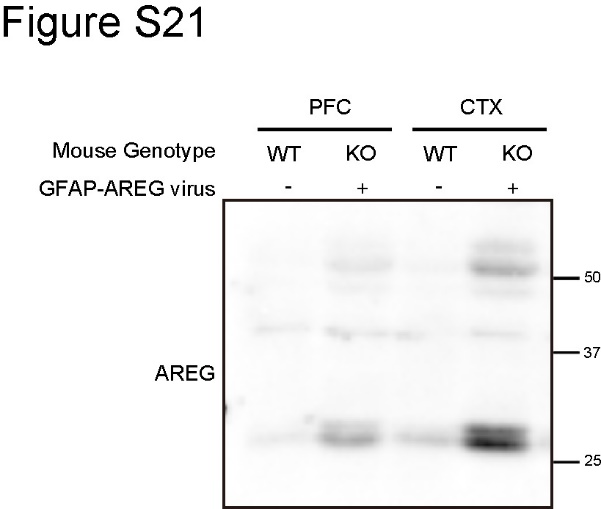


**Supplementary Figure 22:** Immunoblot assay showing the overexpression of amphiregulin in astrocyte cells after administration of GFAP-AREG virus to CRTC3 KO mice.


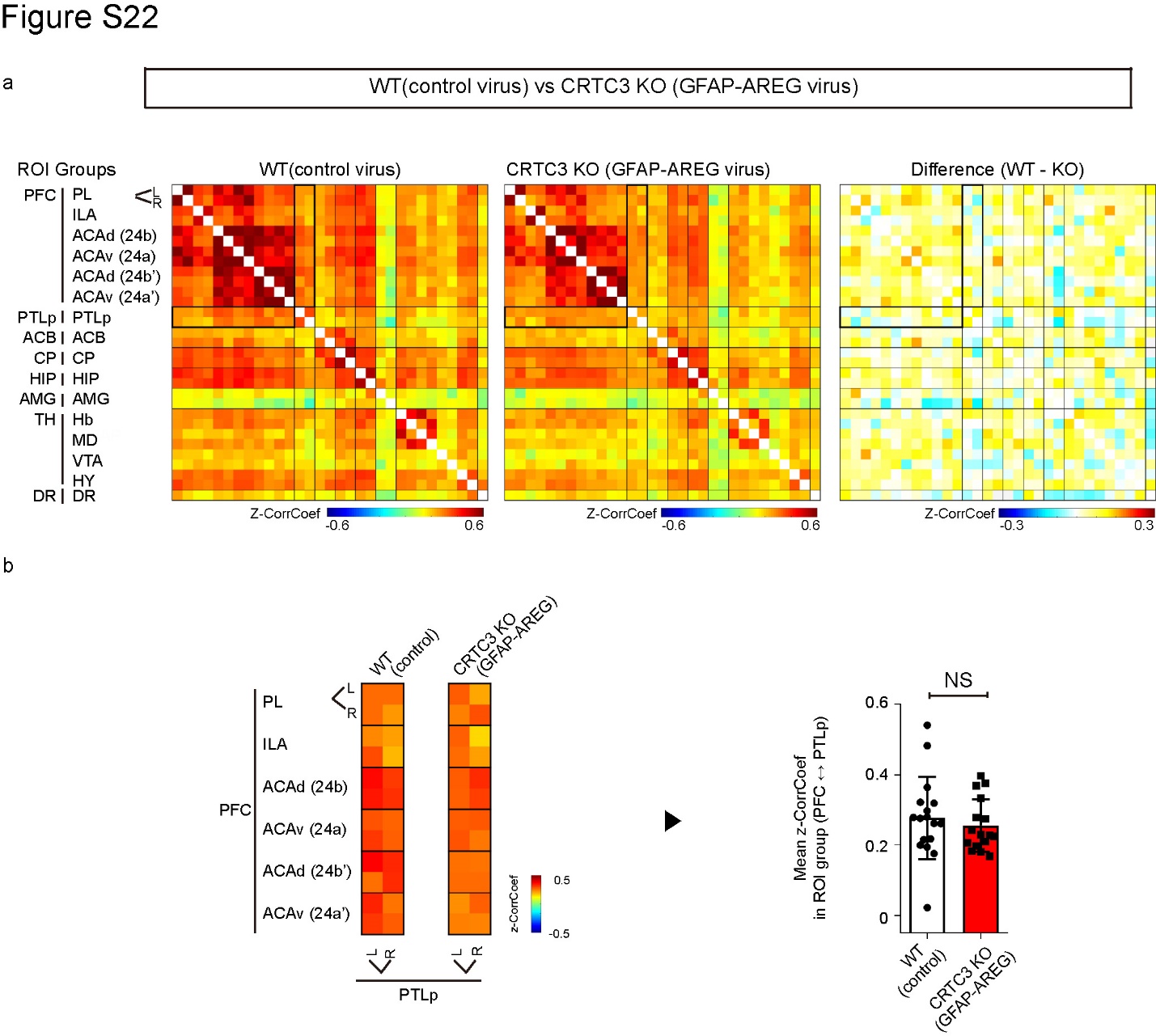


**Supplementary Figure 23:** Resting-state correlation matrix for all pairs of seed ROIs related to the social dominance behavior in the astrocytic expression of AREG virus-injected WT and CRTC3 KO mice.

**a,** The resting-state functional connectivity matrices of social dominance-related brain ROIs from GFAP-AREG virus-injected WT (left) and CRTC3 KO (middle) mice are represented. The mean difference map is represented (right). **b,** Compared with WT, GFAP-AREG virus-infected CRTC3 KO mice show no significant difference for the mean z-CorrCoef between the ROI groups of the prefrontal cortex (PFC) and posterior parietal cortex. L: left and, R: right hemispheres, WT (n = 17), KO (n = 16), Unpaired t-test, p = ns, Error bars, SEM.


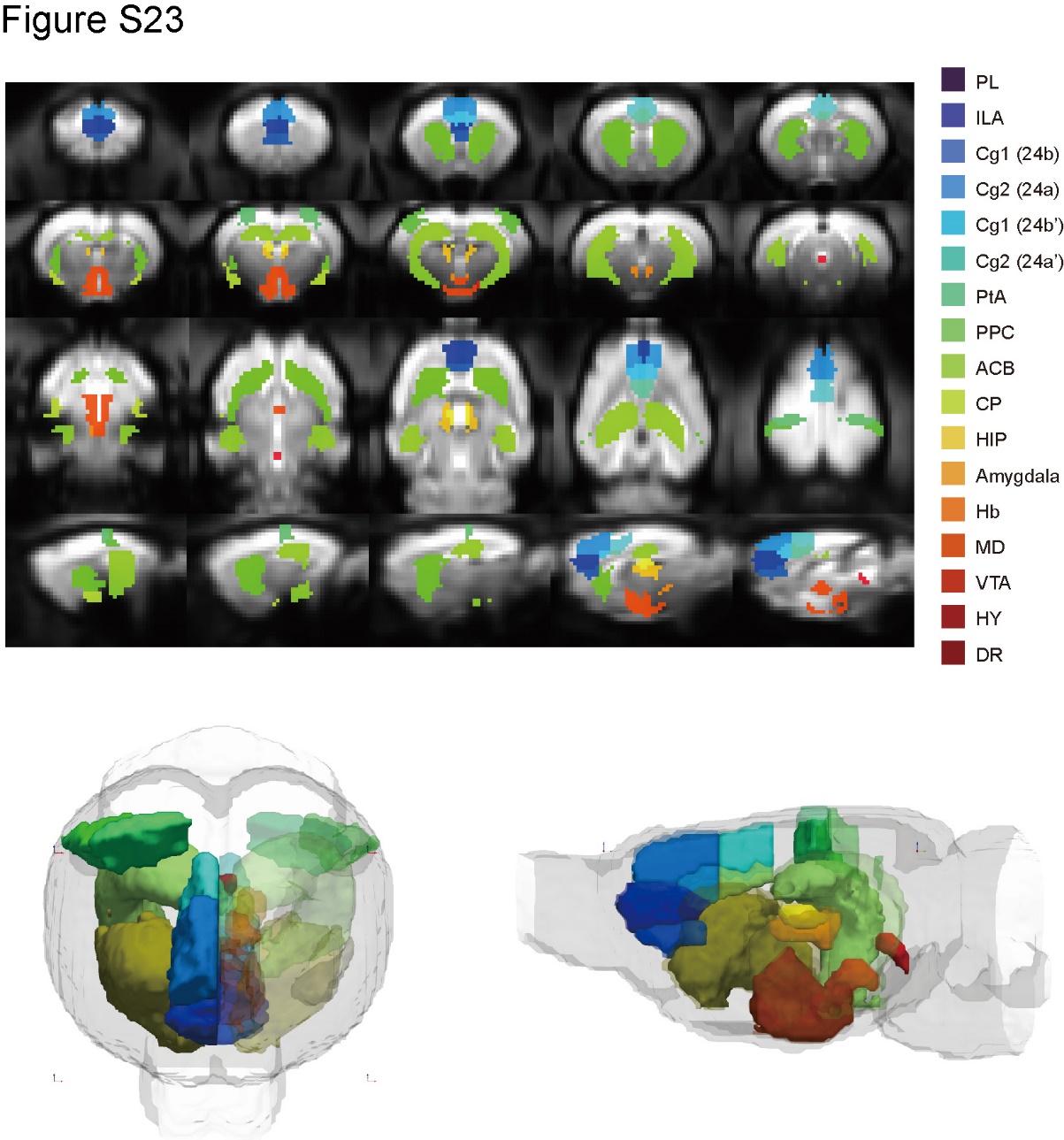


**Supplementary Figure 24:** Regions-of-interest (ROIs) definitions in rodent social dominance-related brain areas. Spatial maps of 33 ROIs shown in different colours are overlaid on averaged EPI data (top panel). Representations of 3D-reconstructed maps of the ROIs (bottom panel).


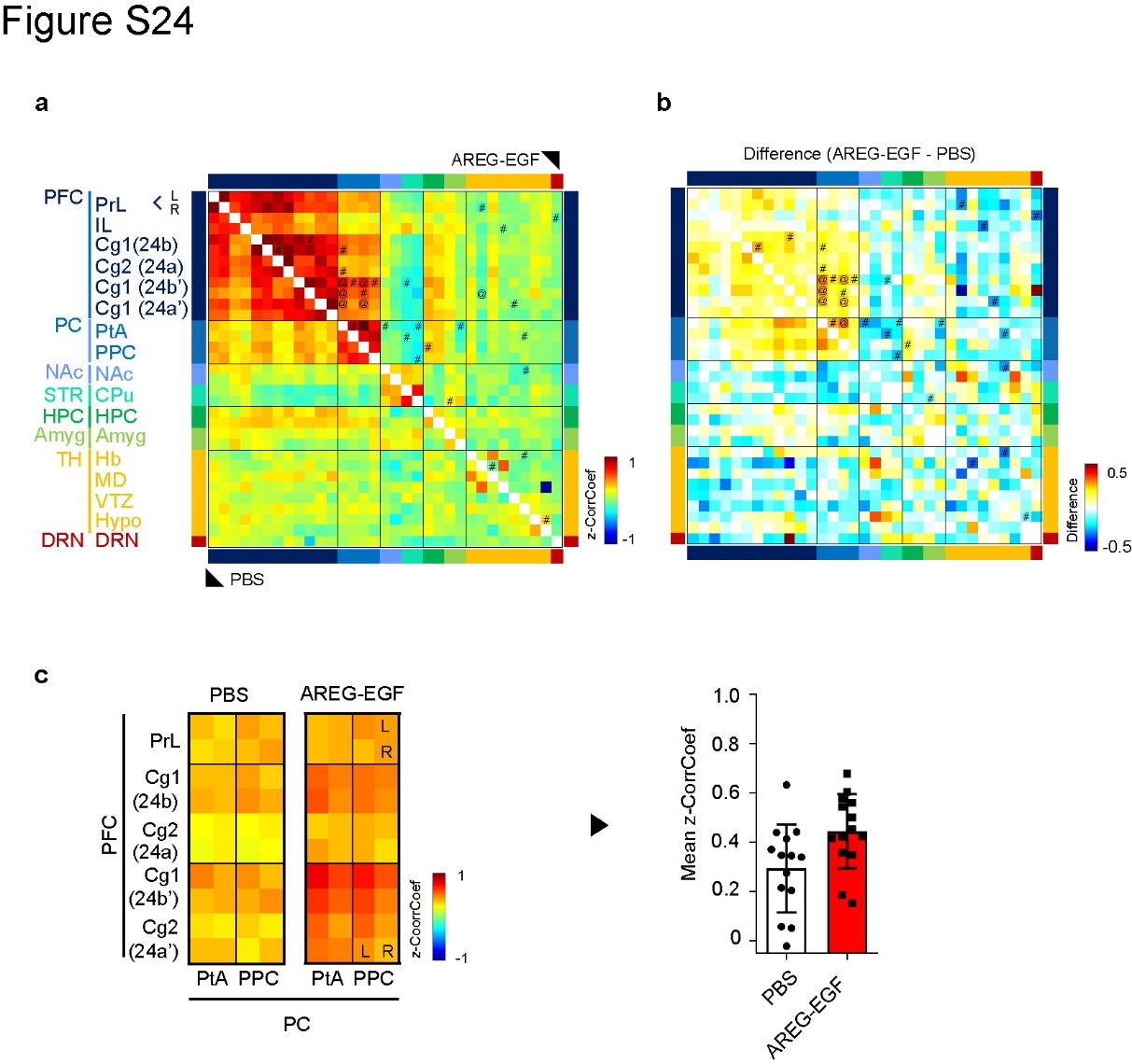


**Supplementary Figure 25:** AREG-EGF treatment upregulates resting-state functional connectivity (rsFC) between the prefrontal cortex (PFC) and the parietal cortex (PC) in rat**.**

**a,** The rsFC matrices of social dominance-related brain ROIs from PBS (left-below) and AREG-EGF (right-upper) groups are shown. **b,** Group difference map (AREG-EGF minus PBS). Student’s t-test, #p<0.05 and @p<0.01. **c,** AREG-EGF treatment increased the mean z-CorrCoef scores across all focused matrices compared with PBS. (see table S1), Student’s t-test, *p<0.05. L: left hemisphere, R: right hemisphere.

**Supplementary Tables**

**Supplementary Table. S1**

Regions-of-interest (ROIs) definitions of mouse social dominance-related brain areas

Region-of-interests (ROI) : ROI groups (*) and ROIs (**)

| PFC, Prefrontal cortex * | L/R (mouse, rat) |
| --- | --- |
| PL, Prelimbic area ** | L/R (mouse, rat) |
| ILA, Infralimbic area ** | L/R (mouse, rat) |
| ACAd(24b), Anterior cingulate area, dorsal anterior part ** | L/R (mouse) |
| ACAv(24a), Anterior cingulate area, ventral anterior part ** | L/R (mouse) |
| ACAd(24b’), Anterior cingulate area, dorsal posterior part ** | L/R (mouse) |
| ACAv(24a’), Anterior cingulate area, ventral posterior part ** | L/R (mouse) |
| Cg1(24b), Primary cingulate cortex anterior part ** | L/R (rat) |
| Cg2(24a), Secondary cingulate cortex anterior part ** | L/R (rat) |
| Cg1(24b’), Primary cingulate cortex posterior part ** | L/R (rat) |
| Cg2(25a’), Secondary cingulate cortex posterior part ** | L/R (rat) |
| PTLp, Posterior parietal association areas *,** | L/R (mouse) |
| PC, parietal cortex * | L/R (rat) |
| PtA, Parietal associative cortex ** | L/R (rat) |
| PPC, Posterior parietal cortex ** | L/R (rat) |
| ACB, Nucleus accumbens *,** | L/R (mouse, rat) |
| CP, Caudoputamen *,** | L/R (mouse, rat) |
| HIP, Hippocampal region *,** | L/R (mouse, rat) |
| AMG, Amygdala nucleus (LA+BLA+BMA+PA) *,** | L/R (mouse, rat) |
| TH, Thalamus * | L/R (mouse, rat) |
| Hb, Habenula (MH+LH) ** | L/R (mouse, rat) |
| MD, Mediodorsal nucleus of thalamus ** | L/R (mouse, rat) |
| VTA, Ventral tegmental area ** | L/R (mouse, rat) |
| HY, Hypothalamus ** | L/R (mouse, rat) |
| DR, Dorsal nucleus raphe *,** | Inter (mouse), L/R (rat) |

**Supplementary Table. S2.**

| **FIGURE NUMBER** | **Statistic method** | **Number** | **Statistic results** | **VALUE** |
| --- | --- | --- | --- | --- |
| 1b | Mann-Whitney test | n = 33 for WT, n = 20 for CRTC3 KO | p = 0.0003 | U = 146.5 |
| 1c | Mann-Whitney test | n = 67 for WT, n = 67 for CRTC3 KO in Mean duration/push n = 67 for WT, n = 67 for CRTC3 KO in # of behaviors/trial | p < 0.0001, p < 0.0001 | U = 1040, U = 928 |
| 1d | Mann-Whitney test | n = 67 for WT, n = 67 for CRTC3 KO in Mean duration/push n = 67 for WT, n = 67 for CRTC3 KO in # of behaviors/trial | p = 0.8908, p = 0.5202 | U = 2218, U = 2011 |
| 1e | Mann-Whitney test | n = 31 for WT, n = 58 for CRTC3 KO in resistance | p = 0.1524 | U = 2738.5 |
| 1f | Mann-Whitney test | n = 31 for WT, n = 58 for CRTC3 KO in retreat | p = 0.1524 | U = 738.5 |
| 1g | Mann-Whitney test | n = 64 for WT, n = 36 for CRTC3 KO in time in warm spot(s) n = 64 for WT, n = 36 for CRTC3 KO in rank in warm spot test | p < 0.0001, p < 0.0001 | U = 552, U = 411.5 |
| 1h | Chi-square | n = 51 for WT, n = 28 for CRTC3 KO in familiar group n = 12 for WT, n = 11 for CRTC3 KO in unfamiliar group | p < 0.0001, p < 0.0001 | 92.48 28.88 |
| 1i | Mann-Whitney test | n = 17 for WT, n = 17 for CRTC3 KO | p = 0.0025 | U = 64.5 |
| 1k | Unpaired t-test | n = 19 for WT, n = 19 for CRTC3 KO | p = 0.0053 | t = 2.967 |
| 2a | Tukey's multiple comparisons test | n = 12 for astrocyte, n = 14 for neuron, n = 8 for microglia | p < 0.0001 for astrocyte vs neuron, p < 0.0001 fot astrocyte vs microglia | q = 11.57, q = 16.14 |
| 2d | Wilcoxon signed rank test | n = 17 for before, n = 17 for after | p < 0.0001 | W = 172 |
| 2e | Kruskal-Wallis test | n = 17 for rank1, n = 17 for rank2 | p < 0.0001 for -5 days rank1 vs -5 days rank2, p < 0.0001 for 53 days rank1 vs 53 days rank2, | 894.4 |
| 2f | Wilcoxon signed rank test | n = 20 for before, n = 20 for after | p < 0.0001 | W = 172 |
| 2h | Unpaired t-test | n = 20 for before, n = 21 for after | p = 0.0225 | t = 2.377 |
| 3b | Tukey's multiple comparisons test | n = 12 for astrocyte, n = 14 for neuron, n = 8 for microglia | p < 0.0001 for astrocyte vs neuron, p < 0.0001 fot astrocyte vs microglia | q = 8.395, q = 7.961 |
| 3c | Unpaired t-test | n = 10 for WT, n = 10 for CRTC3 KO | p = 0.0041 | t = 3.292 |
| 3d | Tukey's multiple comparisons test | n = 6 for 0hr WT, n = 6 for 1hr WT, n = 6 for 2hr WT, n = 6 for 6hr WT, n = 6 for 0hr KO, n = 6 for 1hr KO,  n = 6 for 2hr KO, n = 6 for 6hr KO | p < 0.0001 for 0h:WT vs. 1h:WT p < 0.0001 for 0h:WT vs. 2h:WT p < 0.0001 for 1h:WT vs. 1h:KO p < 0.0001 for 2h:WT vs. 2h:KO | q = 10.35, q = 16.92, q = 12.38, q = 18.19 |
| 3e | Unpaired t-test | n = 6 for Areg-Luc-CTRL, n = 6 for Areg-Luc-FSK,  n = 6 for Areg-Luc-CRTC3,  n = 6 for Areg-Luc-CRTC3-FSK,  n = 6 for Areg-mt-CTRL,  n = 6 for Areg-mt-FSK,  n = 6 for Areg-mt-CRTC3,  n = 6 for Areg-mt-CRTC3-FSK | p = 0.0012 for Areg-Luc-CTRL vs Areg-Luc-FSK, p = 0.0037 for Areg-Luc-CRTC3 vs Areg-Luc-CRTC3-FSK, p = 0.0028 for Areg-mt-CTRL vs Areg-mt-FSK, p = 0.8191 for Areg-mt-CRTC3 vs Areg-mt-CRTC3-FSK | t = 8.229, t = 6.083, t = 6.587, t = 0.2442 |
| 3f | Unpaired t-test | n = 2 for DMSO IgG, n = 2 for FSK IgG, n = 2 for DMSO CRTC3, n = 2 for FSK CRTC3 | p = 0.0355 for DMSO CRTC3 vs FSK CRTC3 | t = 5.163 |
| 4a-left | Kruskal-Wallis test | n = 11 for WT1 (PBS), n = 17 for WT2 (PBS),  n = 17 for KO(AREG) | p = 0.0075 for -5 days WT1 (PBS) vs -5 days WT2 (PBS) p < 0.0001 for -5 days WT1 (PBS) vs -5 days KO (AREG), p = 0.0048 for -5 days WT2 (PBS) vs -5 days KO (AREG) p = 0.001 for 25 days WT1 (PBS) vs 25 days WT2 (PBS), p = ns for 25 days WT1 (PBS) vs 25 days KO (AREG), p = 0.01 for 25 days WT2 (PBS) vs 25 days KO (AREG) | 297 |
| 4a-right | Wilcoxon signed rank test | n = 16 for before, n = 16 for after | p < 0.0001 | W = -136 |
| 4b | Wilcoxon signed rank test | n = 16 for before, n = 17 for after | p = 0.0313 for rank in warm spot(s), p = 0.0129 for time in warm spot(s) | w = -21, w = 103 |
| 4c-left | Kruskal-Wallis test | n = 9 for Rank1 (PBS), n = 17 for Rank2 (PBS),  n = 17 for Rank3(AREG) | p = 0.0033 for -5 days Rank1(PBS) vs -5 days Rank2(PBS) p < 0.0001 for -5 days Rank (PBS) vs -5 days Rank3(AREG), p = 0.0002 for -5 days Rank2(PBS) vs -5 days Rank3(AREG), p = 0.0114 for 25 days Rank1(PBS) vs 25 days Rank2(PBS), p = ns for 25 days Rank1 (PBS) vs 25 days Rank3(AREG), p = ns for 25 days Rank2 (PBS) vs 25 days Rank3(AREG) | 361 |
| 4c-right | Wilcoxon signed rank test | n = 23 for before, n = 23 for after | p < 0.0001 | W = 172 |
| 4d-left | Kruskal-Wallis test | n = 9 for Rank1 (PBS), n = 10 for Rank2 (PBS),  n = 10 for Rank3(AREG-EGF) | p = 0.0033 for -5 days Rank1(PBS) vs -5 days Rank2(PBS) p < 0.0001 for -5 days Rank (PBS) vs -5 days Rank3(AREG), p = 0.0002 for -5 days Rank2(PBS) vs -5 days Rank3(AREG), p = 0.0114 for 25 days Rank1(PBS) vs 25 days Rank2(PBS), p = ns for 25 days Rank1 (PBS) vs 25 days Rank3(AREG), p = ns for 25 days Rank2 (PBS) vs 25 days Rank3(AREG) | 361 |
| 4d-right | Wilcoxon signed rank test | n = 10 for before, n = 10 for after | p = 0.0030 | W = 36 |
| 4e-left | Kruskal-Wallis test | n = 7 for WT1 (PBS), n = 13 for WT2 (PBS),  n = 13 for KO(AREG-EGF) | p = 0.0322 for -5 days WT1 (PBS) vs -5 days WT2 (PBS) p = 0.0002 for -5 days WT1 (PBS) vs -5 days KO (AREG-EGF) p = ns for -5 days WT2 (PBS) vs -5 days KO (AREG-EGF) p = ns for 25 days WT1 (PBS) vs 25 days WT2 (PBS), p = ns for 25 days WT1 (PBS) vs 25 days KO (AREG), p = 0.0461 for 25 days WT2 (PBS) vs 23 days KO (AREG) | 185.9 |
| 4e-right | Wilcoxon signed rank test | n = 8 for before, n = 8 for after | p < 0.0001 | W = 172 |
| 4f-left up | Kruskal-Wallis test | n = 10 for Rank1 (PBS), n = 10 for Rank2 (PBS),  n = 10 for Rank3(AREG-HB) | p = 0.0062 for -5 days Rank1(PBS) vs -5 days Rank2(PBS) p < 0.0001 for -5 days Rank (PBS) vs -5 days Rank3(AREG-HB), p = 0.0062 for -5 days Rank2(PBS) vs -5 days Rank3(AREG-HB), p = 0.0026 for 25 days Rank1(PBS) vs 25 days Rank2(PBS), p < 0.0001 for 25 days Rank1 (PBS) vs 25 days Rank3(AREG-HB), p = 0.0285 for 25 days Rank2 (PBS) vs 25 days Rank3(AREG-HB) | 185.9 |
| 4f-right up | Wilcoxon signed rank test | n = 10 for before, n = 10 for after | p = 0.5 | W = -1 |
| 4f-left down | Kruskal-Wallis test | n = 4 for Rank1 (PBS), n = 4 for Rank2 (PBS),  n = 4 for Rank3 (mEGF) | p = 0.0308 for -5 days Rank1(PBS) vs -5 days Rank2(PBS) p = 0.0025 for -5 days Rank (PBS) vs -5 days Rank3(mEGF), p = ns for -5 days Rank2(PBS) vs -5 days Rank3(mEGF), p = nsfor 25 days Rank1(PBS) vs 25 days Rank2(PBS), p = 0.0028 for 25 days Rank1 (PBS) vs 25 days Rank3(mEGF), p = ns for 25 days Rank2 (PBS) vs 25 days Rank3(mEGF) | 203.8 |
| 4f-right down | Mann-Whitney test | n = 4 for before, n = 4 for after | p = ns | U = 32 |
| 5b | Kruskal-Wallis test | n = 15 for WT (Control), n = 15 for CRTC3 KO(AREG) | p < 0.0001 for -5 daysWT(control) vs -5 days KO(AREG) p = 0.0242 for 37 daysWT(control) vs 37 days KO(AREG) | 278.9 |
| 5c | Wilcoxon signed rank test | n = 15 for before, n = 15 for after | p < 0.0001 | W = 172 |
| 5d | Wilcoxon signed rank test | n = 17 for before, n = 17 for after | p < 0.0001 | W = 172 |
| 5f | Unpaired t-test | n = 19 for Before, n = 16 for After | p = 0.0032 | t = 3.182 |
| 6a | Unpaired t-test | n = 7 for WT, n = 6 for CRTC3 KO | p = 0.0497 | t = 2.204 |
| 6b | Unpaired t-test | n = 12 for WT, n = 7 for CRTC3 KO | p = 0.5721 | t = 0.5761 |
| 6c | Unpaired t-test | n = 16 for WT, n = 8 for CRTC3 KO | p = 0.9405 | t = 0.07553 |
| S1a | Mann-Whitney test | n = 19 for WT, n = 19 for KO | p < 0.0001 | U = 188 |
| S1b | Mann-Whitney test | n = 70 for WT, n = 70 for KO | p = 0.0018 | U = 1749 |
| S2 | Wilcoxon signed rank test | n = 27 for WT, n = 27 for KO | p = 0.0002 | W = -290 |
| S4a | Unpaired t-test | n = 17 for WT, n = 16 for CRTC3 KO | p = 0.0818 for Time in new section, p = 0.5287 for Entry in new section | t = 1.799, t = 0.6372 |
| S4b | Bonferroni's multiple comparisons test, Unpaired t-test | n = 21 for WT, n = 18 for CRTC3 KO | p = ns for latency to target(trial day), p = ns for distance to target(trial day), p = 0.9523 p = 0.939 | t = 0.06036, t = 0.07707 |
| S5a | Tukey's multiple comparisons test | n = 25 for WT, n = 22 for KO | p = ns | 0.683 |
| S5b | Bonferroni's multiple comparisons test | n = 36 for WT, n = 24 for KO | p = ns | 0.2495 |
| S5c | Unpaired t-test | n = 34 for WT, n = 21 for CRTC3 KO | p = 4236 | t = 0.8064 |
| S6a | Tukey's multiple comparisons test | n = 7 for WT, n = 3 for CRTC3 KO | p = ns | 0.0267 |
| S6b | Tukey's multiple comparisons test | n = 14 for Day1 of WT, n = 8 for Day2 of WT, n = 8 for Day1 of KO, n = 3 for Day2 KO | p = ns | 0.1709 |
| S7a | Unpaired t-test | n = 16 for WT, n = 15 for CRTC3 KO | p = 0.3244 for WT vs KO in peanut, p = 0.7639 for WT vs KO in 2MBA | t = 1.003, t = 0.3032 |
| S7b | Mann-Whitney test | n = 19 for WT, n = 17 for KO | p = 0.1215 | U = 0 |
| S9b | Mann-Whitney test | n = 8 for WT, n = 7 for KO | p = 0.0007 | U = 112 |
| S13b | Unpaired t-test | n = 22 for Before, n = 18 for After, | p = 0.7966 | t = 0.2596 |
| S14b | Unpaired t-test | n = 22 for Control, n = 21 for GFAP-Cre, | p = 0.9546 | t = 0.05725 |
| S14d | Unpaired t-test | n = 18 for Control, n = 20 for GFAP-Cre, | p = 0.0379 | t = 2.156 |
| S15a | Unpaired t test | n=12 for Astrocyte, n=12 for Neuron, n=10 for Microglia | p<0.0001 for Astrocyte vs Neuron, p<0.0001 for Astrocyte vs Microglia | t=15.82, t=15.42 |
| S15b | Unpaired t test | n=12 for Astrocyte, n=12 for Neuron, n=10 for Microglia | p<0.0001 for Astrocyte vs Neuron, p<0.0001 for Astrocyte vs Microglia | t=12.94, t=20.08 |
| S15c | Unpaired t test | n=12 for Astrocyte, n=12 for Neuron, n=10 for Microglia | p<0.0001 for Astrocyte vs Neuron, p<0.0001 for Astrocyte vs Microglia | t=27.84, t=29.87 |
| S15d | Unpaired t test | n=12 for Astrocyte, n=12 for Neuron, n=10 for Microglia | p<0.0001 for Astrocyte vs Neuron, p<0.0001 for Astrocyte vs Microglia | t=8.277, t=2.819 |
| S18a | Mann-Whitney test | n=47 for Before, n=47 for After in # of behaviors/trial(push-initiated) n=47 for Before, n=47 for After in # of Mean duration/push(push-initiated) n=47 for Before, n=47 for After in # of behaviors/trial(push-back) n=47 for Before, n=47 for After in # of Mean duration/push(push-back) n=40 for Before, n=26 for Resistance n=40 for Before, n=26 for After in # of Retreat | p<0.0001 for After in # of behaviors/trial(push-initiated), p<0.0001 for After in Mean duration/push(push-initiated), p=ns for After in # of behaviors/trial(push-back), p=ns for After in Mean duration/push(push-back), p=0.0788 for Resistance, p=0.0788 for for Retreat | U=685.5, U=694.5, U=1068, U=1064, U=390.5, U=390.5 |
| S18b | Mann-Whitney test | n=19 for Before, n=19 for After in # of behaviors/trial(push-initiated) n=19 for Before, n=19 for After in # of Mean duration/push(push-initiated) n=19 for Before, n=19 for After in # of behaviors/trial(push-back) n=19 for Before, n=19 for After in # of Mean duration/push(push-back) n=19 for Before, n=9 for Resistance n=19 for Before, n=9 for After in # of Retreat | p=0.1660 for After in # of behaviors/trial(push-initiated), p=0.0137 for After in Mean duration/push(push-initiated), p=ns for After in # of behaviors/trial(push-back), p=ns for After in Mean duration/push(push-back), p=0.0852 for Resistance, p=0.0852 for for Retreat | U=135, U=100.5, U=143, U=144.5, U=54, U=54 |
| S22b | Unpaired t test | n=17 for WT(control), n=16 for KO(GFAP-AREG) | p=0.5483 | t=0.6070 |
| S24c | Unpaired t test | n=14 for PBS, n=14 for AREG-EGF | P=0.0233 | T=2.410 |

Supplementary Videos. S1–S3 are available on line.

**Supplementary Video. S1:** Tube dominance test between control WT and CRTC3 KO mice

**Supplementary Video. S2**: Tube dominance test between control and astrocyte-specific CRTC3 KO mice

**Supplementary Video. S3**: Tube dominance test between amphiregulin infused WT and CRTC3 KO mice.
